# Synchronous PET/MR imaging and visualizing penetration of nanomedicine within tumor based on ^68^Ga-NaGdF_4_ probes

**DOI:** 10.1186/s12951-026-04325-1

**Published:** 2026-04-01

**Authors:** Sitong Wu, Wenyue Li, Qi Yang, Lele Song, Wenpeng Huang, Zhao Chen, Bixiao Cui, Jie Lu, Yinghua Zou, Yi Hou, Lei Kang

**Affiliations:** 1 Department of Radiology, China-Japan Friendship Hospital, Beijing, 100029 China; 2 Department of Nuclear Medicine, Peking University First Hospital, Beijing, 100034 China; 3 Department of Interventional Radiology and Vascular Surgery, Peking University First Hospital, Beijing, 100034 China; 4 College of Materials Science and Engineering, College of Life Science and Technology, Beijing University of Chemical Technology, Beijing, 100029 China; 5 Department of Radiology and Nuclear Medicine, Xuanwu Hospital, Capital Medical University, Beijing, 100053 China

**Keywords:** Synchronous multi-modality imaging, Magnetic resonance imaging, Positron emission tomography, NaGdF_4_ nanoparticles, Penetration

## Abstract

Magnetic resonance imaging (MRI) and nuclear medicine imaging are widely used for cancer detection due to their high soft tissue resolution and sensitivity. Each modality has inherent limitations; therefore, integrated techniques such as positron emission tomography/magnetic resonance (PET/MR) have been developed. However, achieving simultaneous signal enhancement for both PET and MRI in clinical settings remains challenging. In this study, we employed gallium-68 (^68^Ga)-labelled NaGdF_4_@RGD nanoprobes as a model system to establish a synchronous PET/MR imaging strategy for tumor diagnosis. These nanoprobes exhibited suitable radiochemical half-life, and effective tumor-associated targeting, enabling successful PET/MR imaging across multiple tumor models, including hepatoma, renal cancer, and breast cancer *in vivo*. Notably, this imaging strategy allowed time-resolved visualization of nanoprobe distribution and prolonged retention behavior in orthotopic liver tumors during delayed imaging periods, providing imaging-based insights into nanomedicine pharmacodynamics.

**Graphical abstract:**

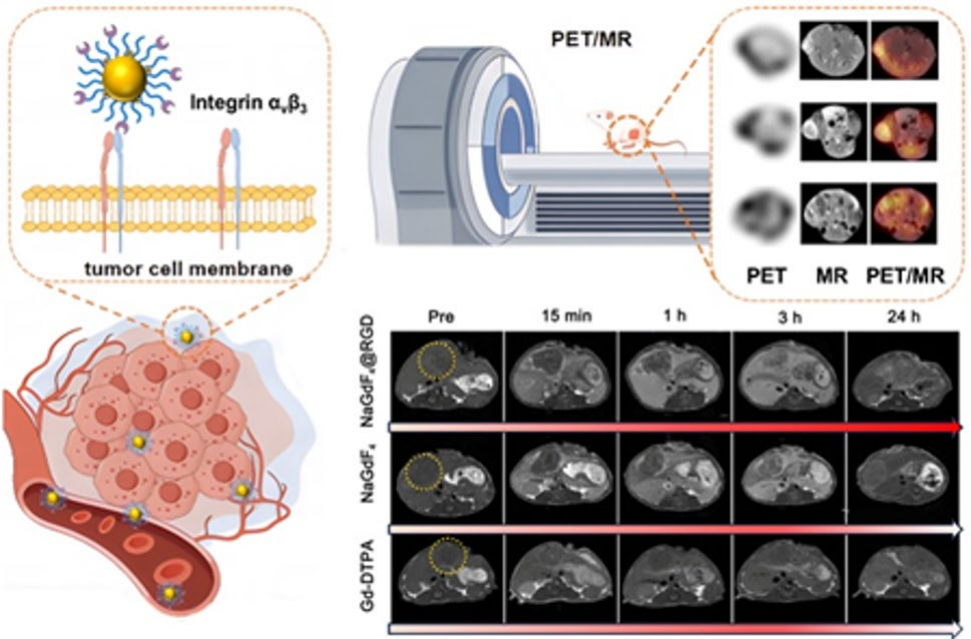

**Supplementary Information:**

The online version contains supplementary material available at 10.1186/s12951-026-04325-1.

## Introduction

Medical imaging techniques, including computed tomography (CT), magnetic resonance imaging (MRI), positron emission tomography (PET), and single-photon emission computed tomography (SPECT), are widely recognized for their immense potential in the diagnosis and therapeutic monitoring of tumors [1]. Nevertheless, a single imaging technique falls short in providing precise tumor diagnosis and is insufficient for acquiring comprehensive information. Multimodal imaging approaches, such as PET/CT or SPECT/CT, integrate the advantages of different imaging modalities, providing simultaneous physiological and morphological information in both preclinical and clinical research settings [2]. For instance, the PET/CT imaging based on ^18^F-FDG has been well established in clinical practice of malignant tumor detection, staging, and response assessment [3].

MRI can provide high spatial resolution of soft tissues, and lack of ionizing radiation enablingto correct PET-induced partial volume effects and to deliver highly detailed, noninvasive anatomical imaging [4]. Therefore, the multi-modality PET/MR imaging, especially the integrated PET/MR, has been developed for high-resolution and high-contrast imaging, coupled with the provision of multiparametric, functional, and quantitative imaging. In early practice, hybrid PET/MRI was achieved by co-administering a mixture of MRI and PET imaging agents with possible high risks to patients, and the mixture failed to ensure precise spatiotemporal correlation between the two imaging modalities due to the distinct biodistribution, pharmacodynamic and pharmacokinetic properties of the imaging agents [5]. Multi-modal contrast agents capable of simultaneously enhancing both PET and MRI signals in clinical practice remain scarce, so the the full potential of PET/MR cannot be fully exploited, and patients may need to undergo multiple injections and scans.

Benefiting from advances in nanotechnology, functional nanomaterials provide versatile platforms for the development of PET/MR imaging probes [1, 6]. Currently, most nanoparticle-based PET/MR contrast agents rely on iron or manganese oxide nanoparticles [7, 8], while Gd is mainly used in the form of small‑molecule contrast agents with potential toxicity risks. Gd‑based nanoparticles for PET/MR remain rare and lack disease‑specific recognition [1, 9], which has limited the development of Gd nanoparticles in the field of PET/MR.

In MR imaging, compared with conventional small-molecule Gd agents, Gd-based nanoparticles can prevent Gd leakage and avoid related toxicity. Moreover, appropriate surface modification prolongs their blood half-life and extends the imaging window [10, 11]. Therefore, Gd-doped inorganic NaGdF_4_ nanoparticles have been widely explored as promising candidates for multimodal imaging systems. In PET imaging, the radionuclide gallium-68 (^68^Ga) features a half-life of ~ 67.6 min that matches the imaging window of nanoprobes, making it a widely used radiotracer [12]. When conjugated with targeting ligands, such as RGD which binds to integrins highly expressed in tumors [13, 14], the nanoprobes enable effective tumor identification [15–17]. However, it is still a challenge to combine the radionuclide ^68^Ga with NaGdF_4_ nanoparticles.

Coordination chemistry represents the prevailing radiolabeling strategy, whereby radionuclides are immobilized on the surface of nanomaterials. This is achieved by introducing a specific chelator on the surface of the nanomaterial in combination with a metal radionuclide, which relies on the strong coordination between the metal radionuclide and the chelator attached to the surface of the nanomaterial [18]. However, in the ligand chemistry approach, modification of the chelating groups may result in alterations to the physicochemical properties of the particles. Additionally, factors such as the shedding of ligands on the surface and *in vivo* transchelation of proteins may compromise the stability of the radiolabelling [19]. An alternative approach is to label the radionuclide directly at the interface between the nanoparticles and the surface ligand, eliminating the need for a chelating agent. The ligands may be simple hydrocarbon chains, peptides or polyethylene glycols. In a recent development, Gao et al. have proposed an anchoring group-mediated radiolabeling method, designated as ligand anchoring group mediated radio labeling (LAGMERAL). As an innovative labelling technique, the LAGMERAL approach employs biocompatible ligands, such as polyethylene glycol (PEG), bearing terminal groups, for instance bisphosphonate (DP), as the chelating agent for the radionuclide. This configuration positions the radionuclide between the bisphosphonate group and an adjacent PEG ligand [20].

**Fig. 1:**
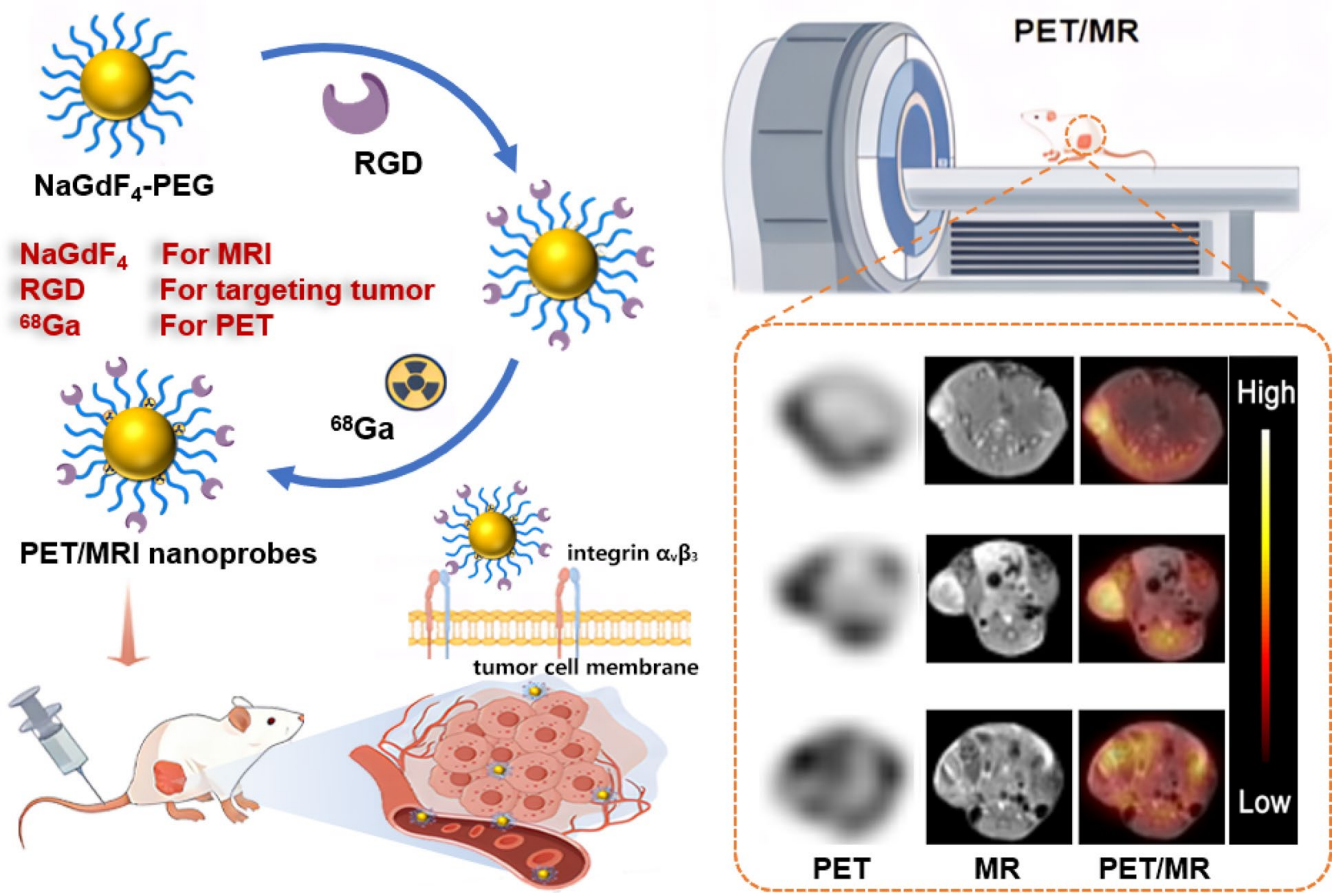
The schematic illustration of the ^68^Ga-NaGdF_4_@RGD nanoprobes

On this basis, we report a synchronous PET/MR imaging strategy based on ⁶⁸Ga-labelled NaGdF_4_@RGD nanoprobes, with particular emphasis on time-resolved visualization of penetration distribution and delayed retention behavior in orthotopic tumor models. As depicted in Fig. 1, an asymmetric PEG ligand (denoted as dp-PEG-mal) bearing diphosphate (DP) and maleimide groups on opposite sides was employed for PEGylation of NaGdF_4_ nanocrystals via the diphosphate group. Notably, the radioactive ^68^Ga was efficiently labelled onto the nanoparticles through the coordination with phosphate groups in PEG ligands, a nanobiotechnological optimization that ensures stable radiolabeling and avoids interference with the probe’s structural integrity. The remaining maleimide groups were used to covalently attach the peptide comprised of cyclo (Arg-Gly-Asp-D-Phe-Cys), which has a strong affinity for the overexpressed integrin α_v_β_3_ on tumor neovascular endothelial cells and tumor cells [21]. The superior *in vivo* tumor targeting capability of the nanoprobes was validated by MR and PET imaging in a murine Hepa1-6 hepatocellular carcinoma model, respectively, while the synchronous PET/MR multimodal fusion imaging performance was confirmed across a series of tumor models.

To a certain extent, this study expands the research on the construction and application of multimodal PET/MR nanoprobes based on Gd-based nanoparticles. Moreover, it should be clarified that the primary contribution of this study does not lie in the development of a fundamentally new RGD ligand or Gd-based nanomaterial. Instead, the novelty of the present work resides in establishing a synchronous, time-resolved PET/MR imaging strategy that enables direct visualization of nanomedicine distribution and delayed retention behavior in orthotopic liver tumors.

## Materials and methods

### Reagents

The following materials were purchased from Sigma-Aldrich: GdCl_3_·6H_2_O, oleic acid (OA), 1-octadecene (ODE), ammonium fluoride (NH_4_F), tris(2-carboxyethyl) phosphine hydrochloride (TCEP), and methyl thiazolyl tetrazolium (MTT). Analytical grade chemicals such as ethanol, cyclohexane, and tetrahydrofuran (THF) were purchased from Sinopharm Chemical Reagent Beijing, Co., Ltd. Polyethylene glycol (PEG) with two phosphate groups at one end of the chain and a maleimide group at the other end (mal-PEG-dp) were customized products provided by Jinan Songren Hightech Co. Ltd. Cyclo [Arg-Gly-Asp-d-Tyr-Lys (COCH_2_CH_2_SH)] (RGD) was purchased from ChinaPeptides (QYAOBIO) Co. Ltd. All reagents for cell culture were obtained from Gibco (ThermoFisher Scientific, Waltham, MA, USA).

### Cell culture and animal tumor model

The murine renal cortical adenocarcinoma cell line Renca and murine triple-negative mammary carcinoma cell line 4T1 were obtained from the American Type Culture Collection, and cultured in RPMI medium 1640 (GIBCO) supplemented with 10% FBS (BI), penicillin and streptomycin (100 U/mL, Hyclone, USA) in an incubator under 5% carbon dioxide atmosphere at 37 °C in a relative humidity of 95%. The murine hepatocellular carcinoma cell line Hepa1-6 was given by the Hepatobiliary and Pancreatic Interventional Treatment Center, The First Affiliated Hospital, Zhejiang University School of Medicine, and cultured in DMEM (Gibco) supplemented with 10% FBS (BI), penicillin and streptomycin (100 U/mL, Gibco, USA) in an incubator under 5% carbon dioxide atmosphere at 37 °C in a relative humidity of 95%. Subcutaneous tumor-bearing models were built by subcutaneous injection of 1 × 10^7^ Renca, 4T1, or Hepa1-6 cells in 6-8-week-old male BALB/c or C57BL/6 mice (purchased from Hua Fukang, Beijing). All animal experiments were approved by Peking University Animal Studies Committee, according to the guidelines for the Care and Use of Research Animals (Peking University, China) (Approval ID J2022100). When the diameter of tumors reached 1 cm, mice were used for subsequent experiments.

The concentration of mouse hepatocellular carcinoma Hepa 1–6 cells was adjusted to 1.0–5.0 × 10^6^ cells/mL using a mixture of phosphate-buffered saline (PBS) and sodium alginate in a 1:1 volume ratio. Intrahepatic tumor-bearing mice were built by intrahepatic injection of 10 µL of the Hepa 1–6 cell suspension into the liver of male C57BL/6 mice. The MRI experiment was performed when the tumor size in the mice liver reached approximately 5–10 mm.

### Relaxivity measurements

The MRI performance of nanomaterials in the current studies was conducted on a 7 T small animal MRI scanner Bruker BioSpec 70/20 USR (Bruker BioSpin MRI, Ettlingen, Germany Co. Ltd.). The aqueous solutions of either NaGdF_4_@RGD particles or gadolinium-diethylenetriaminepentaacetic acid (Gd-DTPA) with a series of Gd^3+^ concentrations were added in 0.2 mL Eppendorf tubes were prepared. The *T*_1_ measurements were as follows: echo time (TE) = 5.01 ms; repetition time (TR) = 300 ms; number of excitations (NEX) = 6. Also, a reference baseline acquisition using a rapid *T*_1_ mapping protocol was obtained. The relaxation properties of the nanoparticles were evaluated using a 7T MRI system. The longitudinal relaxation time (*T*_1_) was measured from the *T*_1_-weighted image of NaGdF_4_@RGD nanoprobes and Gd-DTPA at different concentrations. The relaxation rate (*R*_1_) was obtained by taking the inverse of them and plotting it as a linear correlation between the relaxation rate and the concentration of Gd^3+^. The slope of the correlation corresponds to the molar relaxation rate (*r*_1_).

### MR imaging of subcutaneous tumor *in vivo*

The subcutaneous tumor-bearing mice with mouse hepatoma cell line Hepa 1–6 were intravenously injected with NaGdF_4_@RGD probe, the NaGdF_4_ probe, or Gd-DPTA at a dose of 0.1 mmol Gd^3+^ per kg body weight. MRI images were acquired at different time points, including pre-injection, 15 min, 1 h, 2 h, 3 h, and 4 h post-injection. Axial MRI *T*_1_-weighted sequences and *T*_1_ mapping sequences of the subcutaneous tumor were obtained using a 7T small animal magnetic resonance imaging system (Bruker BioSpec 70/20 USR). The imaging parameters were set as follows: field of view (FOV) = 3.5 × 3.5 cm^2^; matrix size = 256 × 256; slice thickness = 1 mm; echo time (TE) = 5.2 ms; repetition time (TR) = 354.63 ms; number of excitations (NEX) = 1. The parameters for the *T*_1_ mapping sequence were as follows: TR = 461.8-500-1000-1500-3000 ms, TE = 5.95 ms, NEX = 1.0, and matrix size = 100 × 100. The mice were anesthetized by 2% isoflurane delivered *via* a nose cone during the imaging session.

### Small animal PET imaging

Subcutaneous Hepa1-6 tumor–bearing mice were injected intravenously with 18.5 MBq of ^68^Ga-NaGdF_4_ or ^68^Ga-NaGdF_4_@RGD per mouse (*n* = 3), corresponding to an injected nanoparticle mass of 600 µg. Based on these values, the specific activity at the time of injection was approximately 30.8 MBq mg⁻¹. Small animal PET imaging, image reconstruction, and analysis were performed using a Mediso nanoScan^®^PET scanner at 1, 2 and 3 h post-injection. Before each PET scan, mice were anesthetized with isoflurane (2%) and place in the scanner in a prone position. The regions of interest (ROIs) corresponding to the radioactive uptake areas in the major organs were manually delineated on the PET images. The software automatically calculated the maximum standardized uptake value (SUV_max_) within each ROI.

### Biodistribution of ^68^Ga-nanoparticles

After the last PET/CT scan, heart, liver, spleen, lung, kidney, muscle, bone, tumor, and bladder were collected from Hepa1-6 tumor-bearing mice. In addition, a 1% aliquot of the administered dose was collected as a standard sample for measurement. Radioactivity was measured using a gamma counter. The %ID/g (percentage of injected dose per gram) was calculated as the organ (tissue) counts divided by the injected dose and the organ (tissue) weight multiplied by 100.

### PET/MR multimodal synchronous imaging

Several subcutaneous tumor-bearing mice in mice were used to further validate the ability of the nanoprobe to perform synchronous PET/MR imaging. All imaging was performed with the United Imaging uPMR 790 PET/MR scanner.

The subcutaneous tumor-bearing mice with Hepa 1–6, Renca, and 4T1 were selected. A fat-suppressed 3D *T*_1_-weighted MRI sequence scan was performed as pre-injection imaging data. The mice were then divided into two groups and received intravenous injections of ^68^Ga-NaGdF_4_ or ^68^Ga-NaGdF_4_@RGD, with each mouse receiving a dose of Gd^3+^ at 0.1 mmol/kg and a radioactive dose of approximately 30.8 MBq mg⁻¹. PET and fat-suppressed 3D *T*_1_-weighted MRI images were acquired synchronously at 1, 2, and 3 h after injection, with each acquisition lasting a total of 20 min.

PET images in this study were reconstructed using time-of-flight (TOF) technology with the following reconstruction parameters: three-dimensional mode using the ordered subset expectation maximization (OSEM) algorithm with 20 subsets and four iterations. Scatter, random and attenuation corrections were included in the scan. PET images were reconstructed into a 192 × 192 matrix with a slice thickness of 1.6 mm. For *T*_1_-weighted MRI, a fast gradient-echo planar imaging sequence was used with the parameters: TR = 5.7 ms, TE = 2.43 ms, flip angle = 9°, slice thickness = 1.2 mm, matrix size = 240 × 240, voxel size = 0.5 mm × 0.5 mm × 1.2 mm, NEX = 1. PET/MR PET attenuation correction was based on atlas-based MRI attenuation correction combined with the Dixon water-fat separation method. Original image data were reconstructed using MRI attenuation correction and the OSEM algorithm. Following the generation of fused images, ROIs for tumor and muscle radioactivity uptake at different time points were manually delineated on PET images, with SUV_max_ calculated automatically by the software. The ratio of tumor to muscle SUV_max_ values is reported as the SUV ratio.

### Fluorescent immunohistochemistry

Immunofluorescent staining was performed on Hepa 1–6 orthotopic HCC and paracancerous tissues using standard protocols to visualize CD31 expression. The primary antibody used was rabbit anti-mouse CD31 antibody (1:100, ABclonal), and the secondary antibody used was fluorescently labeled goat anti-rabbit IgG (1:300, ABclonal). Photographs were taken using a fluorescence microscope.

### MR imaging of tumor in situ

Intrahepatic tumor-bearing mice were anesthetized and injected with NaGdF_4_@RGD, NaGdF_4_ probe, or Gd-DTPA *via* the tail vein at a dose of 0.1 mmol Gd^3+^ per kg body weight. MRI was performed on a 7T animal MRI instrument (Bruker Biospec) using a saturation-recovery spin-echo imaging sequence. The imaging parameters were set as follows: FOV = 3.5 × 3.5 cm^2^, matrix size = 256 × 256, slice thickness = 1 mm, TR = 461.8-500-1000-1500-3000 ms; TE = 5.2 ms; NEX = 6.0. *T*_1_ maps were calculated by fitting the TR-dependent signal intensity changes to a single exponential function on a pixel-wise basis. The mice were anesthetized with 2% isoflurane delivered *via* a nose cone during the imaging sessions. Images were acquired before injection and at 15 min, 1 h, 2 h, 3 h, 4 h, and 24 h after injection. The animals were then removed from the magnet to recover from anesthesia.

### Chlorophosphonazo (III) staining assay

Nine intrahepatic tumor-bearing mice were divided into three groups. Then *via* tail vein the NaGdF_4_@RGD or NaGdF_4_ nanoparticles was injected by 0.1 mmol Gd^3+^ per kg body weight. The third group was given an equivalent volume of PBS as a control. Four h after injection, the tumors were dissected, and both the tumor and adjacent non-tumorous tissues were stained with Chlorophosphonazo (III) dye to visualize the gadolinium content within the tumor tissues.

### Statistical analysis

Experimental results are presented as mean ± standard deviation. Statistical analyses of data were performed using GraphPad Prism 8.0 and SPSS software. The Mann-Whitney U test was used to analyze non-normally distributed data for differences between groups. Student’s t-test and ANOVA were used to compare and analyze between groups for normally distributed data. No statistical significance is indicated by *P* > 0.05 (ns). *P* < 0.05 indicates statistical significance, * *P* < 0.05; ** *P* < 0.01; *** *P* < 0.001.

**Fig. 2:**
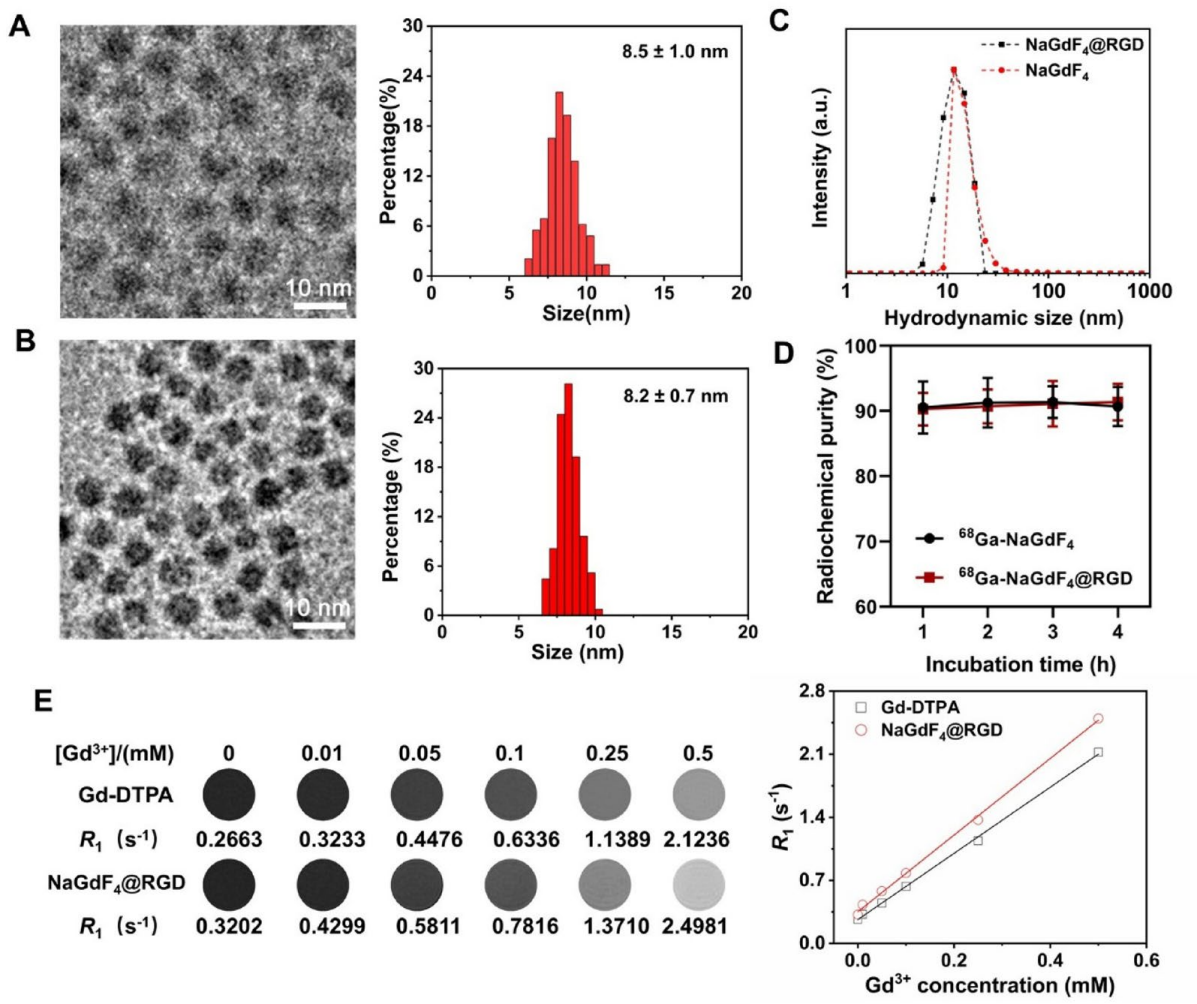
Characterization of nanoprobes. (**A**) TEM images and histograms of the (mal-PEG-dp)-coated NaGdF_4_ nanoparticles; (**B**) TEM images and histograms of the as-prepared NaGdF_4_@RGD nanoprobes; (**C**) Hydrodynamic size profile of the (mal-PEG-dp)-coated NaGdF_4_ and NaGdF_4_@RGD nanoprobes; (**D**) Stability analysis of ^68^Ga-NaGdF_4_@RGD and ^68^Ga-NaGdF_4_ nanoparticles; (**E**) *R*_1_ relaxivity of aqueous solutions containing NaGdF_4_@RGD nanoprobes or Gd-DTPA with different concentrations of Gd^3+^. Data are mean **±** SD. *P < 0.05, **P < 0.01 and ***P < 0.001. ns, not significant

## Results and discussion

### Synthesis and characterization of ^68^Ga- NaGdF_4_@RGD nanoprobes

The NaGdF_4_ nanoparticles were synthesized following the previous high temperature thermal decomposion protocol. After ligand exchange, the NaGdF_4_@RGD nanoprobes were prepared *via* Michael addition reaction between the maleimide groups on the nanoparticle surface and the thiol groups of RGD peptides. As shown in Fig. 2A and B, both NaGdF_4_ and NaGdF_4_@RGD nanoprobes were narrowly dispersed, and the sizes were 8.5 ± 1.0 nm and 8.2 ± 0.7 nm, respectively. DLS analysis was carried out to characterize the aqueous dispersion of the resultant nanoparticles. As shown in Fig. 2C, the size distributions of both NaGdF_4_ and NaGdF_4_@RGD nanoprobes in water exhibit a single peak with a peak position of 11.6 nm, indicating that the nanoparticles did not aggregate during the polypeptide coupling process.

To investigate the possibility of the application of ^68^Ga-NaGdF_4_@RGD for PET imaging, the nanoparticles labeled with ^68^Ga were characterized using thin-layer paper chromatography. Both ^68^Ga-NaGdF_4_@RGD and ^68^Ga-NaGdF_4_ had high labeling rates of 87.2% and 86.8%, respectively. The LAGMERAL method efficiently and quickly labels radionuclide ^68^Ga with inorganic nanoparticles. The labeling process and the repeated ultrafiltration do not have a significant effect on the relaxation properties and the hydrodynamic size of the nanoparticles due to their excellent colloidal stability. Additionally, the labeling rate can be optimized by adjusting the delivery ratio of radionuclide to nanoparticles, reaction time, and other factors [22]. As shown in Fig. 2D, the radiochemical purity of ^68^Ga-NaGdF_4_@RGD and ^68^Ga-NaGdF_4_ remained above 90% after co-incubation in 10% FBS at 37 °C for 4 h, indicating excellent *in vitro* stability in serum. These results suggest that NaGdF_4_@RGD is more stable in labeling ^68^Ga by LAGMERAL method. Although direct *in vivo* radiolabel stability measurements were not performed, the LAGMERAL strategy employed in this study has been previously demonstrated to provide strong coordination between ⁶⁸Ga and phosphate-containing ligands on nanoparticle surfaces [23], resulting in high radiochemical stability under physiological conditions.

The magnetic properties of the nanoparticles were evaluated using a 7T MRI. As shown in Fig. 2E, the *r*_1_ of Gd-DTPA and NaGdF_4_@RGD nanoprobes were 3.67 mM^-1^·s^-1^ and 4.25 mM^-1^·s^-1^, respectively, indicating the potential of NaGdF_4_@RGD as a *T*_1_-weighted MRI contrast agent.

### *In vitro* safety analysis and cellular uptake

**Fig. 3:**
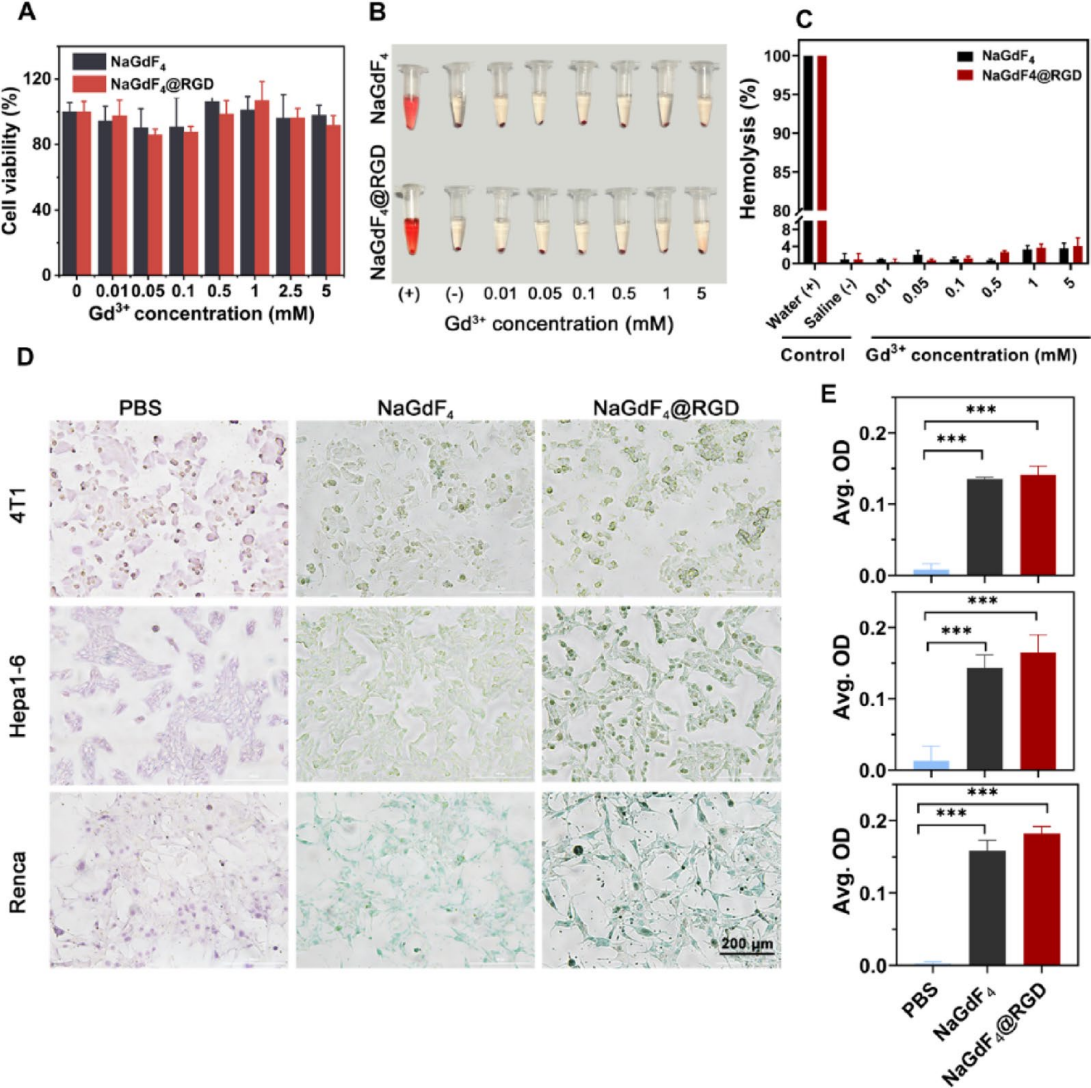
*In vitro* biosafety and targeting assessment of nanoprobes. (**A**) Cell viabilities in NaGdF_4_ and NaGdF_4_@RGD as a function of Gd^3+^ concentration; (**B**) Optical photographs of RBCs co-cultured with pure water (+), 0.9% saline (-), NaGdF_4_, or NaGdF_4_@RGD nanoprobes for 4 h and centrifuged; (**C**) Haemolytic ratio of nanoparticles as a function of Gd^3+^ concentration; (**D**) Photomicrographs and (**E**) optical density of chlorophosphonazo (III) staining of three mouse cell lines co-incubated with PBS, NaGdF_4_ nanoparticles, or NaGdF_4_@RGD nanoprobes for 12 h. Data are mean **±** SD. *P < 0.05, **P < 0.01 and ***P < 0.001. ns, not significant. Scale bar = 200 μm

The viability of Hepa1-6 cells was assessed using CCK-8. As shown in Fig. 3A, there was no statistically significant difference in cell viability between NaGdF_4_ or NaGdF_4_@RGD co-incubated cells as concentration of Gd^3+^ increased from 0.01 to 5 mM (*P* > 0.05). The results showed that NaGdF_4_ and NaGdF_4_@RGD nanoprobes were non-toxic within the tested Gd concentration range. Other studies have also suggested that NaGdF_4_ is not cytotoxic when the Gd^3+^ concentration is below 5 mmol/L, which supports our conclusions [24].

A haemolytic ratio higher than 5% is considered indicative of significant RBC damage [25]. As shown in Fig. 3B, the hemolytic phenomena of these nanodots were hardly visible. the hemolysis percentage was less than 5% even incubated after 4 h at a high concentration of the nanodots (5 mmol/L) (Fig. 3C). There was no statistically significant difference in hemolysis rates between the NaGdF_4_ and NaGdF_4_@RGD nanoprobes. The results indicate that the coupling of RGD peptide did not affect the hemolysis rate of the nanoparticles. This indicates that the particles did not have significant biotoxicity within the given concentration range, ensuring the safety of subsequent *in vivo* experiments.

The renal and liver cancers with high expression levels, were selected for further studies, and 4T1 breast cancer cell line with low expression level serves as negative control.

The cell affinity of nanoparticles was verified by staining assay after co-incubation with different cells. As shown in Fig. 3D, the control group without Gd^3+^ was stained with a chlorophosphonazo (III) solution and the cells were pinkish purple. The experimental group that was co-incubated with rare earth nanoparticles stained with chlorophosphonazo (III) solution showed blue-green cells. The cells co-incubated with NaGdF_4_@RGD nanoprobes were darker, indicating the NaGdF_4_@RGD nanoprobes had a higher affinity for the cells. For quantitative analysis, we calculated the optical densities of the staining results of different cells using Image-Pro Plus software (Fig. 3E). The optical densities of the staining results of the three types of cells co-incubated with NaGdF_4_@RGD and NaGdF_4_ nanoparticles were significantly higher than those of the control group, with statistically significant differences (*P* < 0.001). Cells co-incubated with NaGdF_4_ nanoparticles had higher optical densities than those co-incubated with NaGdF_4_@RGD nanoprobes, but there was no statistically significant difference (*P* > 0.05). The insignificance of the results was primarily attributed to the low sensitivity of chemical staining in detecting gadolinium content within cells.

When co-incubated with NaGdF_4_@RGD, the Renca cell line exhibited the darkest color, followed by Hepa 1–6, and finally 4T1. As shown in Figure S3, there was a statistically significant difference in staining optical density between Renca and 4T1 cells (*P* < 0.05). It is important to note that this evaluation indicating a positive correlation between the cellular expression level of integrin α_v_β_3_ and the degree of NaGdF_4_@RGD nanoprobes uptake by the cells.

**Fig. 4:**
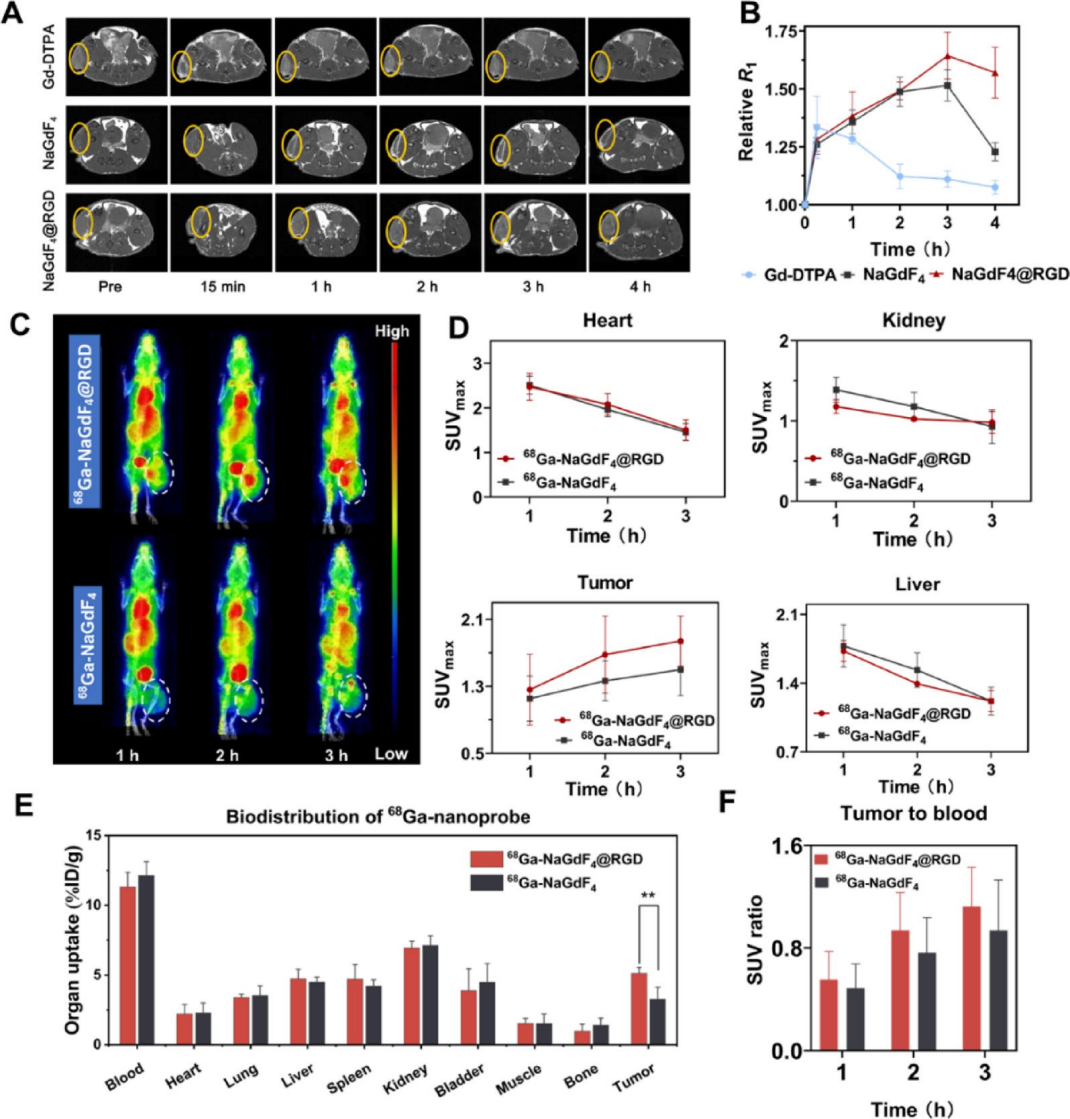
PET and MR imaging in subcutaneous tumor-bearing mouse models. (**A**) Axial images of MRI *T*_1_ enhancement sequence of Hepa1-6 xenografts after injection of NaGdF_4_@RGD, NaGdF_4_, or Gd-DTPA. (**B**) The ratio of *R*_1_ at various time points after nanoprobe injection to the *R*_1_ before injection in subcutaneous tumors. (**C**) The PET images of Hepa 1–6 xenografts after injection of ^68^Ga-NaGdF_4_@RGD or ^68^Ga-NaGdF_4_ are shown in Maximum intensity projection (MIP) format. The tumors are indicated by dashed circles. (**D**) Time-activity curves for different organs were calculated based on ROI analysis of PET imaging. (**E**) The biodistribution results obtained 3 h after the injection of ^68^Ga-NaGdF_4_@RGD or ^68^Ga-NaGdF_4_ are presented. (**F**) The ratio of radioactive uptake in the tumor compared to that in the blood. Data are mean **±** SD. *P < 0.05, **P < 0.01 and ***P < 0.001. ns, not significant

### *In vivo* MRI and PET imaging of intraperitoneal tumor xenografts

On the basis of successful *in vitro* experiments, NaGdF_4_@RGD nanoprobes were used in the following *in vivo* MRI experiments for detecting Hepa1-6 tumors in 6–8 week-old male BALB/c mice, and the corresponding NaGdF_4_ nanoparticles and Gd-DTPA were used as negative controls. A comparison was then made with the enhancement effect of the clinical contrast agent Gd-DTPA (Fig. 4A and B). Over time, the subcutaneous tumor signals of the two experimental groups of mice increased gradually, reaching a peak after 3 h. The Relative *R*_1_ of the tumor sites in NaGdF_4_ and NaGdF_4_@RGD groups was 1.52 ± 0.07、1.64 ± 0.10, respectively. After 3 h, the signal intensities began to decrease. Conversely, in mice injected with Gd-DTPA, the signal at the tumor site was highest only 15 min after injection, with a Relative *R*_1_ of 1.33 ± 0.13. Thereafter, the signal intensity gradually decreased due to the rapid metabolism and short blood circulation time. At 3 h after injection, the tumor Relative *R*_1_ was 1.11 ± 0.03, which was significantly lower than that of the NaGdF_4_@RGD and NaGdF_4_ groups (*P* < 0.001). Apart from the higher Relative *R*_1_ at the tumor site of control mice 15 min after injection, the Relative *R*_1_ at the tumor site of mice in the two nanoparticle experimental groups was higher than that of the control group at the remaining time points. This indicates that nanoparticles have a longer imaging window period and are suitable as contrast agents for PET/MR.

In the NaGdF_4_ group, the signal intensity in the peritumoral regions is higher than that within the tumor core. This observation may be attributed to limitations of the enhanced permeability and retention (EPR) effect imposed by tumor-associated biological barriers, including leaky but heterogeneous vascular endothelium and a dense extracellular matrix (ECM) [26]. As a result, nanoparticles penetrate into tumor tissue is relatively slow, leading to preferential accumulation in peritumoral regions and reflecting restricted penetration redistribution. In addition, interstitial fluid pressure (IFP) is a critical factor influencing the EPR effect [27, 28].With increasing tumor malignancy, IFP within tumor tissues approaches microvascular fluid pressure, thereby diminishing the pressure gradient required for convective transport. This process restricts nanoparticle extravasation through endothelial gaps and reduces the efficiency of passive accumulation mediated by the EPR effect. In comparison, the NaGdF_4_@RGD group exhibits higher signal intensity within tumor regions, indicating enhanced penetration and retention of the RGD-functionalized nanoprobes.

Furthermore, PET imaging serves to validate the tumor-targeting capabilities of nanoparticles. As shown in Fig. 4C and D, there was a continuous increase in radioactivity uptake in Hepa1-6 subcutaneous tumors 1–3 h post-injection, and the tumor radioactivity uptake in the ^68^Ga-NaGdF_4_@RGD group was significantly greater than that in the ^68^Ga-NaGdF_4_ group. At the 3 h following intravenous injection, the SUV_max_ of the Hepa1-6 tumors in the ^68^Ga-NaGdF_4_@RGD group and ^68^Ga-NaGdF_4_ group were 1.84 ± 0.30 and 1.50 ± 0.31, respectively. Additionally, radioactivity uptake at the tumor site increased from 1 to 3 h post-injection, while decreasing in other organs such as blood, kidney, and liver. This indicates the exceptional tumor-targeting ability of ^68^Ga-NaGdF_4_@RGD nanoprobes.

The biodistribution results at 3 h post-injection were generally consistent with the PET imaging observations (Fig. 4E). Tumor uptake of ⁶⁸Ga- NaGdF_4_@RGD reached 5.12 ± 0.42%ID/g, which was higher than that of the non-targeted ⁶⁸Ga- NaGdF_4_ group (3.27 ± 0.87%ID/g, *P* < 0.01). This difference suggests enhanced tumor accumulation of the RGD-functionalized nanoprobes in the Hepa1-6 tumor model.

From 1 to 3 h post-injection, the tumor-to-blood SUV ratios increased over time for both groups (Fig. 4F), reflecting progressive clearance from the circulation. The ⁶⁸Ga- NaGdF_4_@RGD group exhibited numerically higher tumor-to-blood SUV ratios than the non-targeted group at all examined time points. At 3 h post-injection, the tumor-to-blood SUV ratios were 1.12 ± 0.31 for ⁶⁸Ga- NaGdF_4_@RGD and 0.94 ± 0.40 for ⁶⁸Ga- NaGdF_4_, respectively.

Tumor-to-liver ratios (Figure S1) and tumor-to-muscle ratios (Figure S2) were quantitatively compared between the ⁶⁸Ga- NaGdF_4_@RGD and non-targeted ⁶⁸Ga- NaGdF_4_ groups at 1, 2, and 3 h post-injection. Although the mean tumor-to-liver ratios and tumor-to-muscle ratios of the targeted group were consistently higher than those of the non-targeted group across the examined time points, statistical analysis revealed no significant differences between the two groups (all *P* > 0.05).

The sample size in this study was relatively small (*n* = 3 per group), which inevitably limits the statistical power to detect modest differences in SUV-based metrics. Therefore, the present quantitative comparisons should be interpreted as exploratory rather than definitive.

Moreover, quantitative PET analysis of nanoparticle tracers is subject to inherent methodological limitations. Unlike small-molecule radiotracers, nanoparticles typically exhibit prolonged blood circulation, complex clearance pathways, and non-equilibrium tissue distribution, all of which complicate the SUV measurements. spillover from highly perfused organs such as the liver [29] further constrain the sensitivity of SUV-based comparisons, particularly in small cohorts.

These results suggested the specificity and affinity of NaGdF_4_@RGD for binding to tumors. The delayed tumor-associated PET signals observed at later imaging time points are inconsistent with the pharmacokinetics of free ⁶⁸Ga ions, which typically display rapid blood clearance and limited tumor retention [30]. Moreover, previous studies have demonstrated the *in vivo* radiolabeling stability of ⁶⁸Ga-labeled NaGdF_4_-based nanoparticles [23]. Taken together, these considerations further support that the observed PET signals predominantly originate from the radiolabeled nanoprobes rather than from dissociated radionuclide.

Taken together, while the present study demonstrates consistent trends toward enhanced tumor accumulation and retention of ⁶⁸Ga- NaGdF_4_@RGD, the quantitative PET results should be interpreted in conjunction with biodistribution data and MRI findings. Future studies with larger cohorts and optimized quantitative imaging protocols will be required to more precisely resolve the contribution of active targeting to nanoparticle biodistribution.

### PET/MR multimodal synchronous imaging

**Fig. 5:**
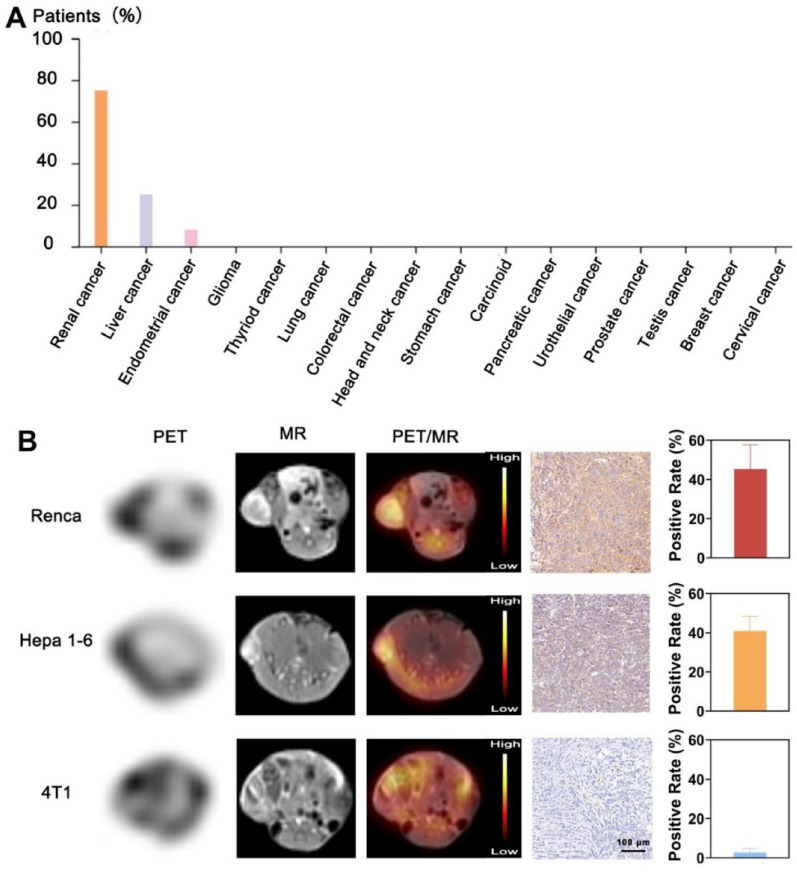
The PET/MR multimodal simultaneous imaging in the three tumor models. (**A**) The expression of integrin β_3_ in various tumor tissues. The data is represented as the percentage of individuals exhibiting high and medium expression levels based on immunohistochemistry, relative to the total number of individuals evaluated. (**B**) from left to right: MRI, PET, and fused images of tumor sites in three types of tumor-bearing mice 3 h after injection of ^68^Ga-NaGdF_4_@RGD; immunohistochemical staining images and quantitative analysis of the positive area for integrin α_v_β_3_ in immunohistochemical sections of the three cancer tissues. * *P* < 0.05; ** *P* < 0.01; *** *P* < 0.001

According to the HPA database, the expression of integrin β_3_ protein is higher in cancers of the kidney and liver (Figure S3), and lower in cancers of the breast. Moreover, we employed animal models to corroborate the correlation between the expression level of integrin α_v_β_3_ and the efficacy of PET/MR imaging in these three tumours. As depicted in Fig. 5A, the MRI, PET, and combined fusion images of subcutaneously tumor-bearing mice (Renca, Hepa 1–6, 4T1) were captured at various time points after injection with ^68^Ga-NaGdF_4_@RGD. A significant enhancement in the MRI *T*_1_ signal is observed at the tumor sites in Renca and Hepa 1–6 over time. The MRI signal intensity and the uptake of radioactivity at the tumor sites suggested a progressive increase over time., reaching its peak 3 h after injection(Figure S4-S6).

The 4T1 tumors exhibited an increase in MRI *T*_1_ signal post-injection. Nevertheless, the enhancement is less pronounced compared to Renca and Hepa 1–6 tumors. In PET imaging, radioactive uptake at the tumor sites also increases over time, but remains relatively low compared to the background. The presence of background uptake in the PET images causes tumor localization and boundary definition to appear blurred. However, the fusion of MRI images can improve the accuracy of tumor detection and localization due to its high spatial resolution. When the two modalities are fused, the radioactive uptake signals on the PET images align with the tumor locations on the MRI images, demonstrating spatial correspondence between PET signal localization and MRI-defined tumor anatomy, thereby improving confidence in tumor identification despite the limited anatomical resolution of PET alone.

Immunohistochemistry was utilized to evaluate the expression of integrin α_v_β_3_ in three types of mouse cancer cell lines: Hepa 1–6, Renca, and 4T1. As shown in Fig. 5B, the percentages of positive area for the Renca and Hepa 1–6 cell lines were more than 40% for integrin α_v_β_3_, while no significant expression of integrin α_v_β_3_ was observed in the 4T1 cell line. The results of the PET/MR imaging showed that the Renca tumors, which had the highest level of integrin α_v_β_3_ expression, had the most superior tumor targeting effect. In contrast, the 4T1 tumors, with the lowest expression of integrin α_v_β_3_, showed lower radioactive uptake. The SUV ratios for Renca, Hepa 1–6, and 4T1 tumors at 3 h post-injection was 7.91 ± 2.17, 6.18 ± 1.51, and 3.50 ± 1.48, respectively (Figure S7). PET lacks anatomical detail but is highly sensitive. When the three tumor models were compared, there was a correlation between the intensity of the radioactivity uptake, and the expression level of integrin α_v_β_3_ in the tumors.

Other studies have also reported a significant correlation between the expression levels of integrin α_v_β_3_ in tumor cells and the degree of uptake of radiolabeled RGD imaging agents in patients with melanoma and sarcoma [31]. In cases such as head and neck squamous cell carcinoma, where integrin α_v_β_3_ is mainly present in the tumor neovascular system rather than in the tumor cells themselves [32], the PET signal intensity correlates with the level of integrin α_v_β_3_ on the tumor vasculature. PET/MR imaging based on ^68^Ga-NaGdF_4_@RGD can be used as a non-invasive tool to visualize and quantify tumor angiogenesis in such tumors. This method shows promise for future applications in screening patients who are suitable for anti-angiogenic therapies. It is important to note that the early effect of anti-angiogenic treatment is to prevent further tumor growth, and significant reductions in tumor size may not be immediately evident. Therefore, measuring tumor size *via* imaging is not an ideal method for evaluating therapeutic efficacy [33]. As an early assessment tool for the efficacy of anti-angiogenic treatments, RGD-based PET/MR imaging offers clear advantages. Additionally, since the expression level of integrin α_v_β_3_ positively correlates with the proliferative and metastatic capabilities of tumors, the ^68^Ga-NaGdF_4_@RGD nanoprobe shows promise for assessing tumor malignancy. Overall, the study suggest that tumor radiotracer uptake can reflect integrin α_v_β_3_ expression levels. The ^68^Ga-NaGdF_4_@RGD probe shows promise for non-invasively localizing and quantifying tumor integrin α_v_β_3_ expression. This provides critical information for early detection, staging, evaluating therapeutic effects, and predicting tumor prognosis.

### Penetration of NaGdF_4_@RGD nanoprobe in orthotopic hepatocellular carcinoma

**Fig. 6:**
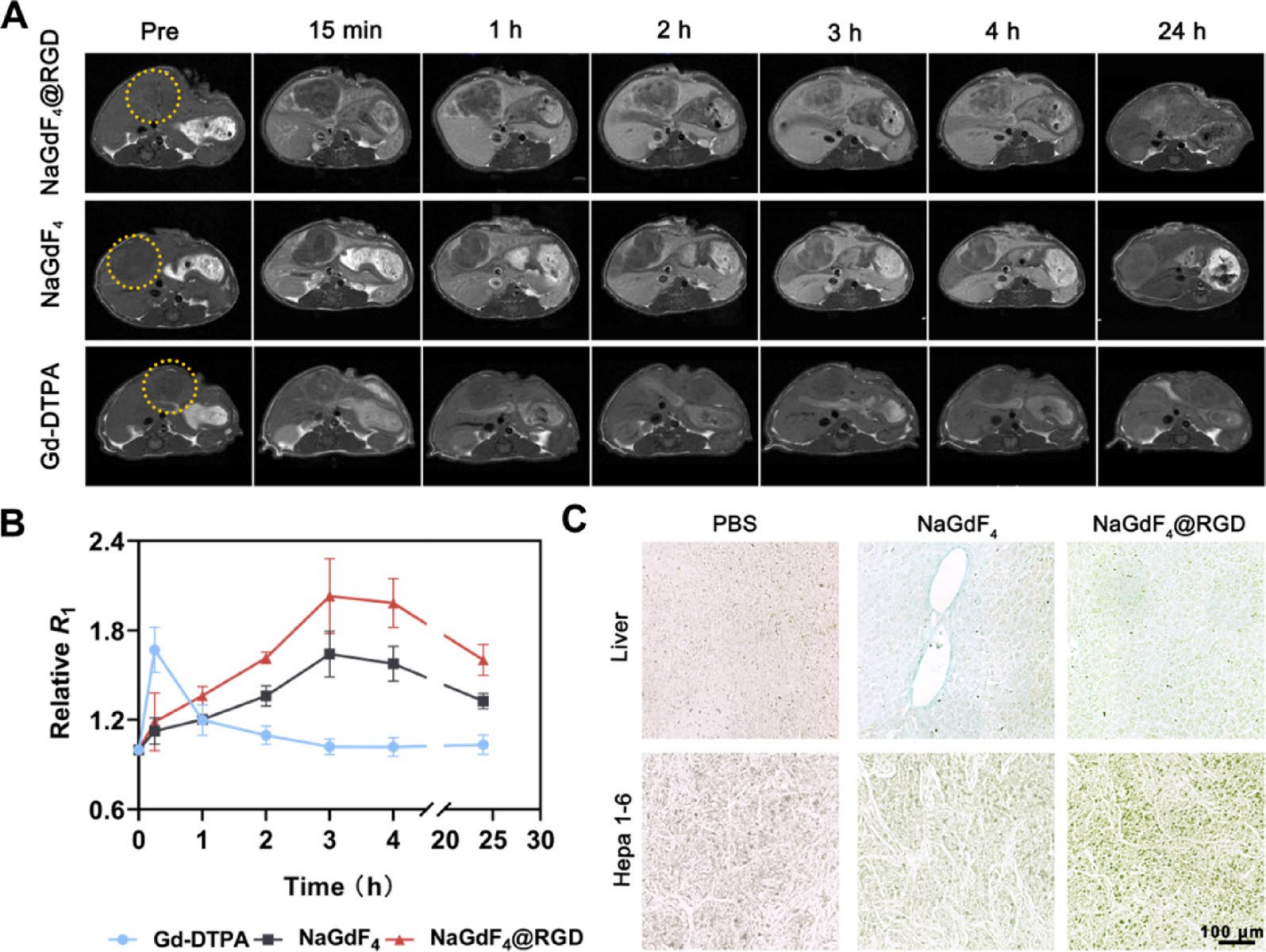
The MR imaging and analysis of in situ tumor-bearing mouse models. (**A**) axial MRI *T*_1_-weighted images of the liver of Hepa 1–6 orthotopic liver cancer mice before and at various time points after the tail vein injection of NaGdF_4_@RGD, NaGdF_4_ nanoprobes, or Gd-DTPA. The yellow circles indicate the tumor areas. (**B**) the ratio of *R*_1_ after injection to *R*_1_ before injection at different time points for the orthotopic tumors in the mice. (**C**) the results of chlorophosphonazo (III) staining of orthotopic tumor and normal liver tissues 4 h after injection of PBS, NaGdF_4_, or NaGdF_4_@RGD nanoprobes. Data are mean **±** SD. *P < 0.05, **P < 0.01 and ***P < 0.001. ns, not significant

As shown in **Figure S8**, the results of the mouse dissection and the pathological sections confirmed that we have successfully established an orthotopic Hepa 1–6 liver cancer mouse model. Axial *T*_1_-weighted MRI of the liver orthotopic tumors were acquired using a 7.0T animal MRI system after injection of NaGdF_4_@RGD, NaGdF_4_, or Gd-DTPA. As shown in Fig. 6A, after injection of Gd-DTPA, the *T*_1_ signal of the orthotopic liver cancer in mice peaked after 15 min, with the corresponding relative *R*_1_ of 1.67 ± 0.15 (Fig. 6B). The intensity of the tumor signal subsequently decreased rapidly, returning almost to the pre-injection level by 3 h after injection, with the relative *R*_1_ of 1.02 ± 0.05. However, the comparison of the liver and tumor signals is not obvious, leading to omission of tumor easily. This is because that Gd-DTPA is a small molecule MRI contrast agent, which can rapidly metabolize from the body through the kidneys, with a short blood half-life [34, 35].

Different from small molecule MRI contrast agent, in mice injected with NaGdF_4_@RGD and NaGdF_4_ nanoparticles, both the signal in tumor and liver signal revealed a gradually rising trend and reached the maxium at 3 h post-injection. This is attributed to the appropriate blood half-life time and targeting capability of nanoprobes [36, 37]. It is interesting to note that, the signal within orthotopic hepatocellular carcinoma exhibited a steadily growing tendency as the liver signals increased, although it shows hypointensity compared to the surrounding parenchyma in the hepatobiliary phase, which is similar to the enhanced MR images in other studies, [38–40], However, different from the quick disappearance of the obvious comparison between tumors and livers, and even at 24 h post-injection, the tumor signal intensity is still higher than that of the pre-injection level, while the liver signal intensity is recovered, resulting in a ‘signal reversal’ phenomenon. In addition, from 15 min to 24 h postinjection, the signal intensities of tumor in mice treated with NaGdF_4_@RGD nanoprobes, were consistently higher than those of the controls. The most notable difference was observed between the 3 and 4 h post-injection (Fig. 5B), indicating enhanced penetration and sustained signal presence of NaGdF_4_@RGD nanoprobes compared with non-targeted controls.

**Fig. 7:**
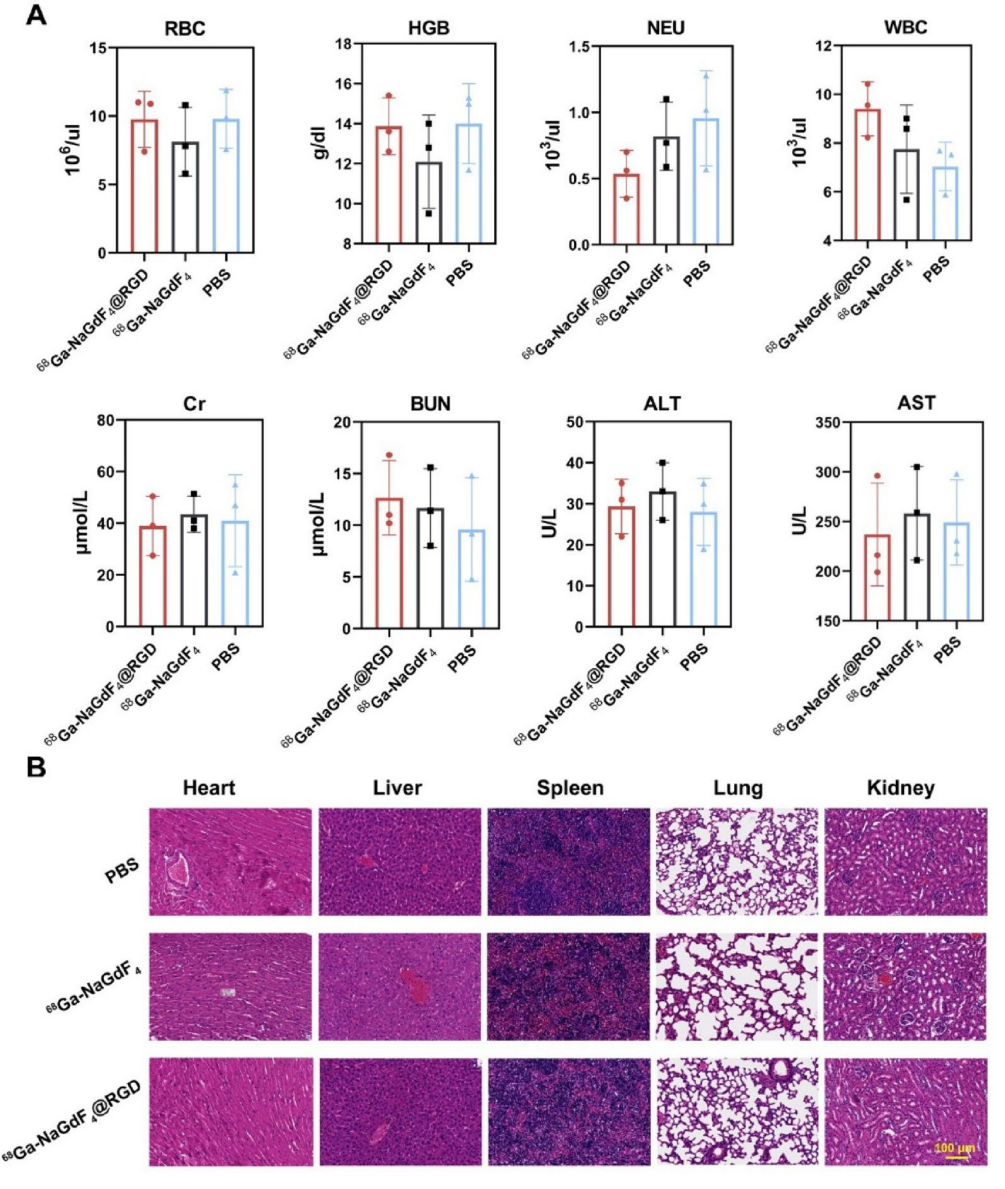
*In vivo* biosafety analysis of nanoprobes. (**A**) Hematology and biochemistry levels 7 days after injection of ^68^Ga-NaGdF_4_@RGD, ^68^Ga-NaGdF_4_, or PBS.Abbreviations: BUN, Blood urea nitrogen; Cr, creatinine ; ALT, alanine aminotransferase; AST, aspartate aminotransferase; RBC, red blood cell; WBC, white blood cell; PLT, platelet; HBG, hemoglobin .(**B**) H&E staining results of major organs two weeks post-injection. Data are mean **±** SD. *P < 0.05, **P < 0.01 and ***P < 0.001. ns, not significant

As depicted in Figure S9, both tumor and liver tissues express CD31, however, tumor tissues exhibit a significantly higher expression level, indicating increased vascular density and angiogenic activity in hepatocellular carcinoma, consistent with previous reports [41, 42]. Physiologically, the progressive reduction of portal tracts during hepatocarcinogenesis leads to a gradual decrease in both arterial and portal venous blood supply to tumor nodules. Meanwhile, the formation of newly developed, unpaired arteries markedly increases arterial perfusion [43].

As a result, during the arterial phase, the local concentration of contrast agents within liver tumor tissues is substantially higher than that in surrounding normal liver tissues.

During the portal venous phase, when contrast agents are predominantly supplied through the portal vein system, normal liver tissues receive a larger proportion of blood flow, whereas tumor tissues experience a relative reduction in contrast agent concentration. Consequently, imaging reveals a pronounced signal decrease in tumor regions compared with normal liver tissues at this stage. With increasing time post-injection, nanoprobes gradually penetrate into the interior of the tumor and simultaneously were uptake of nanoprobes by hepatocytes. Notably, owing to the high expression level of integrin α_v_β_3_ in orthotopic tumors (Figure S10), RGD-functionalized nanoprobes tend to exhibit enhanced penetration and retention within tumor tissues, which likely contributed to the observed signal reversal at delayed imaging time points.

Signal intensity gradient analysis from the tumor periphery to the core was performed using Image J and is presented in Figure S11. With increasing time post-injection, the signal from the tumor periphery to the core showed a gradual increasing trend. At 24 h, the signal intensities in the peripheral and central tumor regions became similar, demonstrating the progressive penetration of nanoprobes into the tumor interior. Owing to RGD modification, the nanoprobes exhibited longer penetration and retention within the tumor compared with the control group.

This imaging behaviour reflects the combined effects of prolonged blood circulation, EPR-mediated accumulation, and active receptor-mediated association, and highlights the advantage of synchronous PET/MR imaging in dynamically monitoring nanomedicine distribution behavior in orthotopic liver tumors. Compared with conventional PET/CT or single-modality MRI enhancement, the present imaging strategy provides improved temporal and spatial insight into nanoparticle behavior.

The chlorophosphonazo (III) staining was performed to confirm the distribution of nanoparticles in tissues (Fig. 6C and Figure S12). At 4 h post-injection, no significant difference in optical density (OD) was observed between normal liver tissues in the NaGdF_4_ and NaGdF_4_@RGD groups (*P* > 0.05), indicating that RGD modification did not markedly increase nonspecific hepatic uptake. In contrast, the staining intensity in tumor tissues of the NaGdF_4_@RGD group was significantly higher than that in the NaGdF_4_ group, demonstrating enhanced tumor-associated accumulation following RGD functionalization. Moreover, within the NaGdF_4_@RGD group, tumor tissues exhibited significantly higher staining intensity than the corresponding normal liver tissues, whereas no statistically significant difference (*P* > 0.05) was observed between tumor and liver tissues in the NaGdF_4_ group. These findings suggest that, at 4 h post-injection, RGD-mediated receptor binding substantially enhances nanoprobe retention in tumor regions.

At 24 h post-injection, the residual nanoparticles within liver vessels and hepatocytes were markedly reduced, resulting in recovery of liver signal intensity. In contrast, the prolonged circulation time, combined EPR effect, and active targeting strategy enabled NaGdF_4_@RGD nanoprobes to persist within tumor tissues for an extended duration. Collectively, these observations provide imaging-supported evidence for differential retention behavior between targeted and non-targeted nanoprobes, which is essential for understanding the *in vivo* uptake and clearance characteristics of nanomedicine in liver tumors.

### *In vivo* safety analysis

To assess the long-term *in vivo* toxicity of the nanoparticles, routine blood and biochemical markers are measured. Approximately ^68^Ga-NaGdF_4_@RGD or ^68^Ga-NaGdF_4_ nanoparticles are injected intravenously into mice, and blood is collected 7 days later for testing. As shown in Fig. 7A, there are no statistically significant differences in biochemical markers such as blood urea nitrogen (BUN) and creatinine (Cr) between the experimental group and the control group (*P* > 0.05), indicating that the nanoparticles do not cause significant effects on kidney function. Similarly, the differences in alanine aminotransferase (ALT) and aspartate aminotransferase (AST) between the experimental and control groups are not statistically significant (*P* > 0.05), suggesting that the nanoparticles do not cause significant liver damage. The routine blood indicators of the experimental group mice, including red blood cell (RBC), white blood cell (WBC), platelet (PLT), and hemoglobin (HGB), are all within the normal range, with no significant statistical differences compared to the control group (*P* > 0.05). This suggests that the nanoparticles do not cause inflammation or damage to major organs in mice.

To better comprehend the potential effects of rare earth nanoparticles on living organisms, mice were dissected two weeks after receiving an intravenous injection of nanoparticles. Histological staining studies were then conducted on their major organs. As demonstrated in Fig. 7B, the mice injected with nanoparticles exhibited good integrity in the tissue structure and cell morphology of organs such as the heart, liver, spleen, lungs, and kidneys, with no evident pathological or inflammatory responses when compared to the control group. Based on the results of hematological analysis and organ histological examination, it can be concluded that the physiological functions and health condition of mice injected with ^68^Ga-NaGdF_4_@RGD nanoprobes were not affected. This suggested the excellent biocompatibility of the nanoparticles *in vivo*.

The *in vivo* biosafety evaluation conducted in this study demonstrated favorable short-term biocompatibility of the ⁶⁸Ga- NaGdF_4_@RGD nanoprobes, supporting their suitability as a platform for multimodal imaging applications. It should be emphasized that the relatively high injected Gd dose employed herein is primarily dictated by the intrinsic sensitivity requirements of MRI. Such dosing strategies are well aligned with established practices in nanoparticle-enhanced MRI, where inorganic Gd-based nanomaterials are commonly administered at higher Gd concentrations to achieve sufficient T₁-weighted signal enhancement *in vivo* [24, 44]. Accordingly, the Gd dosage used in this work reflects methodological necessities rather than excessive or atypical exposure.

The long-term biological fate of NaGdF_4_-based nanomaterials is governed by a combination of particle size, structural stability, and surface chemistry. For nanoparticles within the typical size range used for MR imaging, hepatobiliary processing and sequestration by the mononuclear phagocyte system constitute the predominant clearance pathways, with liver Kupffer cells and splenic macrophages serving as the primary sites of accumulation [29]. Particle size plays a decisive role in determining clearance efficiency and long-term retention. Ultrasmall NaGdF_4_ nanodots with hydrodynamic diameters in the sub-5 nm regime have been shown to undergo efficient renal elimination, thereby markedly reducing long-term hepatic accumulation and improving biosafety profiles. In contrast, larger core–shell or mesoporous NaGdF_4_-based nanoparticles exhibit prolonged circulation and enhanced imaging performance at the cost of increased uptake by the reticuloendothelial system [45, 46]. These observations highlight the inherent trade-off between imaging sensitivity and clearance efficiency, which represents a central design consideration for translational nanoprobes.

In addition to size effects, structural robustness is critical for minimizing the release of free Gd^3+^ ions under physiological conditions. Core–shell architectures and interfacial coordination strategies have been demonstrated to effectively confine lanthanide ions within the crystal lattice, thereby enhancing *in vivo* stability and reducing potential toxicity [47].

In the present study, the NaGdF_4_ nanoprobes possess a hydrodynamic size of approximately 8 nm. The particle size used in this work represents a design that favors prolonged blood circulation and enhanced imaging sensitivity.

Importantly, NaGdF_4_ nanocrystals exhibit high lattice stability, and the confinement of Gd^3+^ within the crystalline matrix significantly reduces the likelihood of free ion release under physiological conditions. This structural feature, which has been further reinforced in core–shell NaGdF_4_ systems, is considered a key factor underlying the favorable *in vivo* biosafety profile reported for rare-earth fluoride nanoprobes [47, 48]. In addition, PEGylation improves colloidal stability and reduces nonspecific protein adsorption, thereby mitigating rapid macrophage-mediated clearance and contributing to more controlled biodistribution.

## Conclusions

In summary, a PET/MR dual-modality imaging system based on ⁶⁸Ga-NaGdF_4_@RGD nanoprobes was established using a ligand anchoring group-mediated radiolabeling strategy. The radiolabeling of ⁶⁸Ga is efficient and operationally straightforward, supporting its potential applicability in PET/MR imaging studies. Both *in vitro* and *in vivo* evaluations suggested acceptable biosafety of the rare-earth-based nanoprobes.

Importantly, this imaging strategy enabled synchronized PET/MR acquisition and accurate multimodal image fusion across multiple tumor models, providing both time-dependent and spatially resolved information on nanoparticle distribution. By leveraging the high soft-tissue contrast and longitudinal capability of MRI, we achieved noninvasive visualization of nanoparticle uptake and tumor-associated accumulation *in vivo*.

The progressive and sustained signal enhancement observed within orthotopic liver tumors, together with the newly incorporated quantitative analysis, supports the occurrence of nanoparticle penetration from the tumor vasculature into the tumor parenchyma at the macroscopic imaging level. Here, the term penetration is intentionally used to describe the overall process by which nanoprobes traverse the vascular–tumor interface and enter tumor tissue, rather than a specific microscopic transport mechanism.

Nevertheless, the current imaging resolution does not allow definitive discrimination among vascular retention, cellular internalization, and interstitial diffusion. Such mechanistic differentiation would require high-resolution histological co-localization or intravital microscopy, which is beyond the scope of this imaging-oriented study.

Importantly, the current study is not intended to position this nanoprobe as an immediately deployable clinical contrast agent, but rather to establish a proof-of-concept strategy for synchronous PET/MR imaging and time-resolved visualization of nanoparticle behavior *in vivo*. The integration of PET and MRI leverages the complementary strengths of both modalities, combining the high sensitivity of PET for early-phase tracer distribution with the superior spatial resolution and delayed imaging capability of MRI. Although ⁶⁸Ga offers practical advantages for early PET imaging, its short physical half-life (67.6 min) inherently limits prolonged radionuclide tracking. In this context, MRI plays a critical role in extending the temporal window for assessing nanoparticle retention. Consistent with this framework, future iterations of the platform may incorporate longer-lived positron-emitting radionuclides, such as ⁶⁴Cu or ⁸⁹Zr, to achieve improved temporal alignment between radionuclide decay and nanoparticle pharmacokinetics.

Looking forward, progression toward clinical applicability is more realistically envisioned as a stepwise optimization process rather than direct translation of the current formulation. Potential development directions include reducing the required Gd dose through enhancement of longitudinal relaxivity, refining nanoparticle size and surface chemistry to improve clearance efficiency, and tailoring radionuclide selection to specific clinical imaging timelines. In this regard, the present work provides a versatile and adaptable imaging framework rather than a single fixed formulation, offering clear pathways for future optimization in response to diverse diagnostic needs.

Overall, this study establishes a coherent experimental and conceptual foundation for the development of next-generation PET/MR nanoprobes, illustrating how rational nanoparticle design and multimodal imaging integration can be synergistically combined to advance the spatiotemporal characterization of tumor-associated nanomedicine behavior.

## Supplementary Information

Below is the link to the electronic supplementary material.

Supplementary Material 1

## Data Availability

The data that support the findings of this study are available from the corresponding author upon reasonable request.

## References

[1] Liu H, Wang R, Gao H, Chen L, Li X, Yu X, et al. Nanoprobes PET/MR Imaging. 2024;7(2):2300232.

[2] Wang L, Tang G, Hu K, Liu X, Zhou W, Li H, et al. Comparison of 68Ga-FAPI and 18F-FDG PET/CT in the Evaluation of Advanced. Lung Cancer. 2022;303(1):191–9. 10.1148/radiol.211424 34981976

[3] Liu Y. FDG PET/CT for metastatic squamous cell carcinoma of unknown primary of the head and neck. Oral Oncol. 2019;92:46–51. 31010622 10.1016/j.oraloncology.2019.03.014

[4] Garcia J, Tang T, Louie AY. Nanoparticle-based multimodal PET/MRI probes. Nanomed (Lond). 2015;10(8):1343–59. 10.2217/nnm.14.224 25955127

[5] de Rosales RT. Potential clinical applications of bimodal PET-MRI or SPECT-MRI agents. J Label Comp Radiopharm. 2014;57(4):298–303. 10.1002/jlcr.3154 PMC4336561 24395384

[6] Kastelik-Hryniewiecka A, Jewula P, Bakalorz K, Kramer-Marek G, Kuźnik N. Targeted PET/MRI Imaging Super Probes: A Critical Review of Opportunities and Challenges. Int J Nanomed. 2021;16:8465–83. 10.2147/IJN.S336299 PMC8733213 35002239

[7] Sabbaghan M, Nigam S, Kasabasic I, Manepalli M, Wang P, Fan J. The Development and Challenges of PET/MRI Dual-Modality Imaging Probes—An. Update. 2025;62(5):1245–59. 10.1002/jmri.29779 PMC12507149 40192185

[8] Lee HY, Li Z, Chen K, Hsu AR, Xu C, Xie J, et al. PET/MRI dual-modality tumor imaging using arginine-glycine-aspartic (RGD)-conjugated radiolabeled iron oxide nanoparticles. J Nucl Med. 2008;49(8):1371–9. 18632815 10.2967/jnumed.108.051243

[9] Truillet C, Bouziotis P, Tsoukalas C, Brugière J, Martini M, Sancey L, et al. Ultrasmall particles for Gd-MRI and (68) Ga-PET dual imaging. Contrast Media Mol Imaging. 2015;10(4):309–19. 25483609 10.1002/cmmi.1633

[10] Zheng XY, Zhao K, Tang J, Wang XY, Li LD, Chen NX, et al. Gd-Dots with Strong Ligand-Water Interaction for Ultrasensitive Magnetic Resonance Renography. ACS Nano. 2017;11(4):3642–50. 28350963 10.1021/acsnano.6b07959

[11] Lu C, Han L, Wang J, Wan J, Song G, Rao J. Engineering of magnetic nanoparticles as magnetic particle imaging tracers. Chem Soc Rev. 2021;50(14):8102–46. 34047311 10.1039/d0cs00260g

[12] N’Guessan É, Bacot S, Raes F, Leenhardt J, Guenard T, Dumas L, et al. Side by side comparison of NOTA and DOTA for conjugation efficiency, gallium-68 labeling, and in vivo biodistribution of anti-mesothelin sdAb A1-His. EJNMMI radiopharmacy Chem. 2025;10(1):54. 10.1186/s41181-025-00380-5 PMC12367608 40833585

[13] Zhang P, Li W, Liu C, Qin F, Lu Y, Qin M, et al. Molecular imaging of tumour-associated pathological biomarkers with smart nanoprobe: From Seeing to Measuring. Explor (Beijing China). 2023;3(6):20230070. 10.1002/EXP.20230070 PMC10742208 38264683

[14] Henssen D, Herings S, Sabri O, Hesse S, van der Kolk A, Arens A, et al. Advances in clinical neuro-oncology research on integrin PET imaging. EJNMMI Rep. 2025;9(1):33. 41016977 10.1186/s41824-025-00270-8 PMC12477096

[15] Li D, Zhao X, Zhang L, Li F, Ji N, Gao Z, et al. (68)Ga-PRGD2 PET/CT in the evaluation of Glioma: a prospective study. Mol Pharm. 2014;11(11):3923–9. 25093246 10.1021/mp5003224 PMC4224544

[16] Li D, Zhang J, Ji N, Zhao X, Zheng K, Qiao Z, et al. Combined 68Ga-NOTA-PRGD2 and 18F-FDG PET/CT Can Discriminate Uncommon Meningioma Mimicking High-Grade Glioma. Clin Nucl Med. 2018;43(9):648–54. 30052597 10.1097/RLU.0000000000002233

[17] Kang F, Wang S, Tian F, Zhao M, Zhang M, Wang Z, et al. Comparing the Diagnostic Potential of 68Ga-Alfatide II and 18F-FDG in Differentiating Between Non-Small Cell Lung Cancer and Tuberculosis. J Nucl Med. 2016;57(5):672–7. 26719378 10.2967/jnumed.115.167924

[18] Gawne PJ, Man F, Blower PJ. Direct Cell Radiolabeling for in Vivo Cell Tracking with PET and SPECT Imaging. Chem Rev. 2022;122(11):10266–318. 35549242 10.1021/acs.chemrev.1c00767 PMC9185691

[19] Xu M, Soliman MG, Sun X, Pelaz B, Feliu N, Parak WJ, et al. How Entanglement of Different Physicochemical Properties Complicates the Prediction of in Vitro and in Vivo Interactions of Gold Nanoparticles. ACS Nano. 2018;12(10):10104–13. 30212621 10.1021/acsnano.8b04906

[20] Gao Z, Hou Y, Zeng J, Chen L, Liu C, Yang W et al. Tumor microenvironment-triggered aggregation of antiphagocytosis (99m) Tc-Labeled Fe(3) O(4) nanoprobes for enhanced tumor imaging in Vivo. Adv Mater. 2017;29(24):1701095. 10.1002/adma.201701095 28402594

[21] Zhao X, Liu C, Wang Z, Zhao Y, Chen X, Tao H et al. Synergistic pro-apoptotic effect of a cyclic RGD peptide-conjugated magnetic mesoporous therapeutic nanosystem on hepatocellular carcinoma HepG2 cells. Pharmaceut. 2023;15(1):276. 10.3390/pharmaceutics15010276 PMC9866545 36678904

[22] Hu Q, Chen Q, Gu Z. Advances in transformable drug delivery systems. Biomaterials. 2018;178:546–58. 29657093 10.1016/j.biomaterials.2018.03.056

[23] Ge J, Chen L, Huang B, Gao Y, Zhou D, Zhou Y, et al. Anchoring Group-Mediated Radiolabeling of Inorganic NanoparticlesA Universal Method for Constructing Nuclear Medicine Imaging Nanoprobes. ACS Appl Mater Interfaces. 2022;14(7):8838–46. 35133124 10.1021/acsami.1c23907

[24] Hou Y, Qiao R, Fang F, Wang X, Dong C, Liu K, et al. NaGdF4 nanoparticle-based molecular probes for magnetic resonance imaging of intraperitoneal tumor xenografts in vivo. ACS Nano. 2013;7(1):330–8. 23199030 10.1021/nn304837c

[25] Kloypan C, Suwannasom N, Chaiwaree S, Prapan A, Smuda K, Baisaeng N, et al. In-vitro haemocompatibility of dextran-protein submicron particles. Artif cells Nanomed Biotechnol. 2019;47(1):241–9. 30663396 10.1080/21691401.2018.1548476

[26] Wang HF, Ran R, Liu Y, Hui Y, Zeng B, Chen D, et al. Tumor-Vasculature-on-a-Chip for Investigating Nanoparticle Extravasation and Tumor Accumulation. ACS Nano. 2018;12(11):11600–9. 30380832 10.1021/acsnano.8b06846

[27] Zhou G, Gao Y, Shi Y, Qiu S, Lin G, Ding X, et al. Design of in vitro biomimetic experimental system and simulation analysis for transvascular transport of nano-preparation. Microvasc Res. 2024;151:104597. 37619888 10.1016/j.mvr.2023.104597

[28] Gao Y, Shi Y, Fu M, Feng Y, Lin G, Kong D, et al. Simulation study of the effects of interstitial fluid pressure and blood flow velocity on transvascular transport of nanoparticles in tumor microenvironment. Comput Methods Programs Biomed. 2020;193:105493. 32408237 10.1016/j.cmpb.2020.105493

[29] Khan IA, Yu T, Yang M, Liu J, Chen Z. A Systematic Review of Toxicity, Biodistribution, and Biosafety in Upconversion Nanomaterials: Critical Insights into Toxicity Mitigation Strategies and Future Directions for Safe Applications. BME Front. 2025;6:0120. 40416504 10.34133/bmef.0120 PMC12099058

[30] Kumar V, Boddeti DK. (68)Ga-radiopharmaceuticals for PET imaging of infection and inflammation. Recent Results Cancer Res. 2013;194:189–219. 22918761 10.1007/978-3-642-27994-2_11

[31] Haubner R, Weber WA, Beer AJ, Vabuliene E, Reim D, Sarbia M, et al. Noninvasive visualization of the activated alphavbeta3 integrin in cancer patients by positron emission tomography and [18F]Galacto-RGD. PLoS Med. 2005;2(3):e70. 15783258 10.1371/journal.pmed.0020070 PMC1069665

[32] Beer AJ, Grosu AL, Carlsen J, Kolk A, Sarbia M, Stangier I, et al. [18F]galacto-RGD positron emission tomography for imaging of alphavbeta3 expression on the neovasculature in patients with squamous cell carcinoma of the head and neck. Clin Cancer Res. 2007;13(22 Pt 1):6610–6. 18006761 10.1158/1078-0432.CCR-07-0528

[33] Chen H, Niu G, Wu H, Chen X. Clinical Application of Radiolabeled RGD Peptides for PET Imaging of Integrin αvβ3. Theranostics. 2016;6(1):78–92. 26722375 10.7150/thno.13242 PMC4679356

[34] Lorusso V, Arbughi T, Tirone P, de Haën C. Pharmacokinetics and tissue distribution in animals of gadobenate ion, the magnetic resonance imaging contrast enhancing component of gadobenate dimeglumine 0.5 M solution for injection (MultiHance). J Comput Assist Tomogr. 1999;23(Suppl 1):S181–94. 10608414 10.1097/00004728-199911001-00023

[35] Tombach B, Heindel W. Value of 1.0- M gadolinium chelates: review of preclinical and clinical data on gadobutrol. Eur Radiol. 2002;12(6):1550–6. 12042967 10.1007/s00330-001-1242-9

[36] Li W, Cheng J, Liu C, Zhang N, Lin H, He F, et al. Shine and darkle the blood vessels: Multiparameter hypersensitive MR angiography for diagnosis of panvascular diseases. Sci Adv. 2024;10(40):eadq4082. 39365870 10.1126/sciadv.adq4082 PMC11451532

[37] Zhang P, Cheng J, Liu C, Li W, Wang Y, Zhang N, et al. Hypersensitive MR Angiography for Diagnosis of Ischemic Stroke and Reperfusion Subarachnoid Hemorrhage. Anal Chem. 2024;96(29):11742–50. 38980807 10.1021/acs.analchem.4c01097

[38] Rao C, Wang X, Li M, Zhou G, Gu H. Value of T1 mapping on gadoxetic acid-enhanced MRI for microvascular invasion of hepatocellular carcinoma: a retrospective study. BMC Med Imaging. 2020;20(1):43. 32345247 10.1186/s12880-020-00433-y PMC7189724

[39] Kang HJ, Lee JM, Jeon SK, Jang S, Park S, Joo I, et al. Intra-individual comparison of dual portal venous phases for non-invasive diagnosis of hepatocellular carcinoma at gadoxetic acid-enhanced liver MRI. Eur Radiol. 2021;31(2):824–33. 32845387 10.1007/s00330-020-07162-4

[40] Joo I, Lee JM, Lee DH, Jeon JH, Han JK, Choi BI. Noninvasive diagnosis of hepatocellular carcinoma on gadoxetic acid-enhanced MRI: can hypointensity on the hepatobiliary phase be used as an alternative to washout? Eur Radiol. 2015;25(10):2859–68. 25773941 10.1007/s00330-015-3686-3

[41] Bösmüller H, Pfefferle V, Bittar Z, Scheble V, Horger M, Sipos B, et al. Microvessel density and angiogenesis in primary hepatic malignancies: Differential expression of CD31 and VEGFR-2 in hepatocellular carcinoma and intrahepatic cholangiocarcinoma. Pathol Res Pract. 2018;214(8):1136–41. 29935812 10.1016/j.prp.2018.06.011

[42] Qian H, Yang L, Zhao W, Chen H, He S. A comparison of CD105 and CD31 expression in tumor vessels of hepatocellular carcinoma by tissue microarray and flow cytometry. Experimental therapeutic Med. 2018;16(4):2881–8. 10.3892/etm.2018.6553 PMC6125829 30214510

[43] Palmer WC, Patel T. Are common factors involved in the pathogenesis of primary liver cancers? A meta-analysis of risk factors for intrahepatic cholangiocarcinoma. J Hepatol. 2012;57(1):69–76. 22420979 10.1016/j.jhep.2012.02.022 PMC3804834

[44] Liu C, Gao Z, Zeng J, Hou Y, Fang F, Li Y, et al. Magnetic/upconversion fluorescent NaGdF4:Yb,Er nanoparticle-based dual-modal molecular probes for imaging tiny tumors in vivo. ACS Nano. 2013;7(8):7227–40. 23879437 10.1021/nn4030898

[45] Xing H, Zhang S, Bu W, Zheng X, Wang L, Xiao Q, et al. Ultrasmall NaGdF4 nanodots for efficient MR angiography and atherosclerotic plaque imaging. Adv Mater. 2014;26(23):3867–72. 24677351 10.1002/adma.201305222

[46] Zhou L, Zheng X, Gu Z, Yin W, Zhang X, Ruan L, et al. Mesoporous NaYbF4@NaGdF4 core-shell up-conversion nanoparticles for targeted drug delivery and multimodal imaging. Biomaterials. 2014;35(26):7666–78. 24929618 10.1016/j.biomaterials.2014.05.051

[47] Chen G, Ohulchanskyy TY, Liu S, Law WC, Wu F, Swihart MT, et al. Core/shell NaGdF4:Nd(3+)/NaGdF4 nanocrystals with efficient near-infrared to near-infrared downconversion photoluminescence for bioimaging applications. ACS Nano. 2012;6(4):2969–77. 22401578 10.1021/nn2042362 PMC3430515

[48] Li W, Cheng J, Zhang X, Wang Y, Wu S, Zhang P, et al. High-Resolution Magnetic Resonance Angiography of Tumor Vasculatures with an Interlocking Contrast Agent. ACS Nano. 2024;18(37):25647–56. 39216081 10.1021/acsnano.4c07533

