# Supplementary material for: Synchronous PET/MR imaging and visualizing penetration of nanomedicine within tumor based on 68Ga-NaGdF4 probes
Source: J Nanobiotechnology. 2026 Apr 1;24:428. doi: 10.1186/s12951-026-04325-1 (PMC13169577; doi:10.1186/s12951-026-04325-1)
Supplement: Supplementary file 1 — Supplementary Material 1 [file 12951_2026_4325_MOESM1_ESM.pdf]

Supporting Information

Synchronous PET/MR Imaging and Visualizing Penetration of Nanomedicine within Tumor Based on ^{68}Ga-$NaGdF_4$ Probes

Sitong Wu[1,2,3, #], Wenyue Li[4, #], Qi Yang[2#], Lele Song[2], Wenpeng Huang[2], Zhao Chen[2], Bixiao Cui[5], Jie Lu[5], Yinghua Zou[3]*, Yi Hou[4]*, Lei Kang[2]**

[1] Department of Radiology, China-Japan Friendship Hospital, Beijing, 100029, China.

[2] Department of Nuclear Medicine, Peking University First Hospital, Beijing, 100034, China.

[3] Department of Interventional Radiology and Vascular Surgery, Peking University First Hospital, Beijing 100034, China.

[4]College of Materials Science and Engineering, and College of Life Science and Technology, Beijing University of Chemical Technology, Beijing 100029, China

[5]Department of Radiology and Nuclear Medicine, Xuanwu Hospital, Capital Medical University, Beijing, 100053, China.

*Corresponding author. Email: kanglei@bjmu.edu.cn; houyi@iccas.ac.cn; pkuzouyhua@163.com; Imaginglu@hotmail.com.

[#] S.W. ， W.L. and Q.Y. contributed equally to this work.

Additional Experimental Details

Chemicals

The following materials were purchased from Sigma-Aldrich: $GdCl_3 \cdot 6H_2O$, oleic acid (OA), 1-octadecene (ODE), ammonium fluoride (NH_4F), tris(2-carboxyethyl) phosphine hydrochloride (TCEP), and methyl thiazolyl tetrazolium (MTT). Analytical grade chemicals such as ethanol, cyclohexane, and tetrahydrofuran (THF) were purchased from Sinopharm Chemical Reagent Beijing, Co., Ltd. Polyethylene glycol (PEG) with two phosphate groups at one end of the chain and a maleimide group at the other end (mal-PEG-dp) were customized products provided by Jinan Songren Hightech Co. Ltd. Cyclo [Arg-Gly-Asp-d-Tyr-Lys ($COCH_2CH_2SH$)] (RGD) was purchased from ChinaPeptides (QYAOBIO) Co. Ltd.

Preparation of $NaGdF_4$@RGD nanoprobes

In a typical preparation, $GdCl_3$ $6H_2O$ (0.371 g, 1 mmol) was dissolved in a mixture of OA (14 mL) and ODE (16 mL). The solution was heated to 150 °C under nitrogen protection to form a homogeneous solution. After cooling to room temperature, a 10 mL methanol solution containing NaOH (0.100 g, 2.5 mmol) and NH_4F (0.148 g, 4 mmol) was slowly added. The product was stirred at 50°C for 30 minutes, then subjected to vacuum at 100 °C for 10 minutes to remove methanol. Subsequently, it was heated to 300 °C under atmospheric pressure using an electric hood and kept under nitrogen protection at the same temperature for 1 h. Finally, the reaction mixture was cooled to room temperature to obtain nanoprobes. The $NaGdF_4$ was precipitated using ethanol, collected through centrifugation, washed multiple times with ethanol, and ultimately redispersed in THF or cyclohexane for subsequent experiments.

10 mg of OA-coated $NaGdF_4$ nanoprobes was mixed with about 100 mg of PEG phosphate ligand. The reaction mixture was then stirred at room temperature overnight. The PEG-coated particles were precipitated with cyclohexane and washed three times with cyclohexane. Finally, the PEG-coated $NaGdF_4$ particles (Hereinafter referred to as $NaGdF_4$) were dried under vacuum at room temperature.

The $NaGdF_4$ nanoprobes coated with (mal-PEG-dp) (8 mg) were mixed with c(RGDfK) (1.8 mg) in Tris-buffered saline (TBS, pH 7.4, 2.5 mM) for 60 minutes, using Michael reaction for subsequent conjugation. The $NaGdF_4$@RGD conjugates was purified by centrifugation for three times at 16000 g in a 30 K MWCO centrifuge unit (Millipore YM-30) to remove the unreacted polypeptide, and then stored at 4°C.

Characterizations

The transmission electron microscopy (TEM) images of the PEG-coated $NaGdF_4$ and $NaGdF_4$@RGD were taken on a JEM-100CXII TEM instrument to reveal the crystal structure of the nanoprobes. ImageJ software was utilized to statistically analyze at least 100 particles to determine the average particle size. The average particle size was determined by counting the size of at least 100 nanoprobes for each sample using ImageJ software. The dynamic light scattering (DLS) measurements were carried out at 298.0 K with Nano ZS (Malvern) equipped with a solid state He–Ne laser (λ = 633 nm) for determining the hydrodynamic size of the $NaGdF_4$@RGD, and monitoring the following bioconjugation reaction. The concentration of Gd^{3+} was determined by using a Thermo ICP 6300 Inductively Coupled Plasma-Atomic Emission Spectroscopy (ICP-AES) instrument.

^{68}Ga labeling of nanoprobes

We performed labeling of ^{68}Ga using the ligand anchoring group-mediated radiolabeling (LAGMERAL) method developed by previous senior researchers[4]. This method involves decorating the surface of inorganic nanoprobes with diphosphonate-polyethylene glycol (dp-PEG), which enables efficient radiolabeling through the diphosphonate groups.

$^{68}GaCl_3$ was produced using a $^{68}Ge/^{68}Ga$ generator (Isotope Technologies Garching GmbH, Germany). The pH of the eluted $^{68}GaCl_3$ solution was adjusted to 4.5 by the addition of NaOAc solution. To label the nanoprobes, 37 MBq of $^{68}GaCl_3$ was added to a clean tube containing the PEG-coated $NaGdF_4$ and $NaGdF_4$@RGD solution (200 g included), and the mixture was heated at 37 °C for 20 minutes. The radiochemical purity was determined using thin-layer chromatography (TLC) with a 0.5 mmol/L $EDTANa_2$ (pH 7.4) solution as the developing agent. Ultrafiltration kits (Millipore, 30K molecular weight cut-off) were used to remove excess $^{68}GaCl_3$. The purified product was configured with PBS as a solution containing 3 mg/ml nanoprobes.

Radiolabeling stability of ^{68}Ga labeling in solution

To evaluate the stability of the complexes obtained, 3.7 MBq of ^{68}Ga-$NaGdF_4$ and ^{68}Ga-$NaGdF_4$@RGD was incubated in fetal bovine serum at 37 °C at the concentration of 3 mg/ml for 1, 2, 3, and 4 h. At the different time points, the radiochemical purity was measured by TLC.

Cell culture and animal tumor model

The murine renal cortical adenocarcinoma cell line Renca and murine triple-negative mammary carcinoma cell line 4T1 were obtained from the American Type Culture Collection, and cultured in RPMI medium 1640 (GIBCO) supplemented with 10% FBS (BI), penicillin and streptomycin (100 U/mL, Hyclone, USA) in an incubator under 5% carbon dioxide atmosphere at 37°C in a relative humidity of 95%. The murine hepatocellular carcinoma cell line Hepa1-6 was given by the Hepatobiliary and Pancreatic Interventional Treatment Center, The First Affiliated Hospital, Zhejiang University School of Medicine, and cultured in DMEM (Gibco) supplemented with 10% FBS (BI), penicillin and streptomycin (100 U/mL, Gibco, USA) in an incubator under 5% carbon dioxide atmosphere at 37°C in a relative humidity of 95%. Subcutaneous tumor-bearing models were built by subcutaneous injection of 1×10^7 Renca, 4T1, or Hepa1-6 cells in male BALB/c or C57BL/6 mice (purchased from Hua Fukang, Beijing). All animal experiments were approved by Peking University Animal Studies Committee, according to the guidelines for the Care and Use of Research Animals (Peking University, China) (Approval ID J2022100). When the diameter of tumors reached 1 cm, mice were used for subsequent experiments.

The concentration of mouse hepatocellular carcinoma Hepa 1-6 cells was adjusted to 1.0 - 5.0×10^6 cells/mL using a mixture of phosphate-buffered saline (PBS) and sodium alginate in a 1:1 volume ratio. Intrahepatic tumor-bearing mice were built by intrahepatic injection of 10 μL of the Hepa 1-6 cell suspension into the liver of male C57BL/6 mice. The MRI experiment was performed when the tumor size in the mice liver reached approximately 5-10 mm.

Cytotoxicity of nanoprobes

Cell Counting Kit-8 (CCK8) assays on Hepa1-6 cells were carried out as follows. Cells were seeded and cultured at a density of 4×10^3/well in 100 μL of medium into 96-well microplates (Corning, USA). The PEG-coated $NaGdF_4$ and $NaGdF_4$@RGD nanoprobes were added to the wells at designed concentrations (0.01, 0.05, 0.1, 0.5, 1, 2.5, 5, and 10 mmol/L), and incubated with the cells for 12 h at 37 °C under 5% CO_2. Then, 10 μL of CCK8 reagent was added to each well and then cultured for 2 h. Every experiment was carried out in triplicate. The absorbance was analysed at 450 nm using a microplate reader using wells without cells as blanks. The absorbance of cells expressed cell proliferation.

Haemolysis test

Mouse heparinized erythrocytes were washed in PBS until the supernatant was clear and colorless. Then, 250 μL of RBCs were diluted with saline solution and mixed with different concentrations of $NaGdF_4$ or $NaGdF_4$@RGD. The distilled water and PBS were employed as the positive and negative control, respectively. After incubation at 37 °C for 4 h, the erythrocyte suspensions were centrifuged. The resulting supernatant was photographed and its absorbance at 541 nm was measured to determine the optical density.

Tumor immunohistochemistry

Through querying the Human Protein Atlas database (HPA, http://www.proteinatlas.org). The HPA could provide IHC results of multiple proteins based on proteomics in both cancer tissues and normal tissues. The expression profiles of the ITGB3 gene at both the mRNA and protein levels in various tissues and cancer tissues was analyzed. In summary, three representative tumor types were selected for detailed analysis: renal cell carcinoma, hepatocellular carcinoma, and breast cancer, based on the descending order of protein expression levels at both the mRNA and protein levels. Tumor samples were collected from Hepa 1-6, Renca, and 4T1 subcutaneous tumor-bearing mouse models, as well as from the Hepa 1-6 orthotopic liver cancer model and adjacent tissues. The expression level of integrin β_3 was investigated in these samples using immunohistochemistry. Scanned histological sections were acquired and Image J image analysis software was used for quantitative assessment of the positively stained areas. The percentage of positive area was utilized as the readout for immunohistochemical staining results.

Cellular uptake assay

Hepa1-6, Renca, and 4T1 cells were co-incubated with $NaGdF_4$@RGD and $NaGdF_4$ nanoprobes at different concentrations for 12 h. After co-incubation, the cells were washed with PBS and fixed with 4% paraformaldehyde. Then, the cells were stained with chlorophosphonazo (III) for 0.5 h to evaluate the binding of nanoprobes to different cell types. A control group of cells that were not co-incubated with nanoprobes

was also included. The optical density (OD) of the stained cell images was calculated using the image processing software Image-Pro Plus.

Blood analysis and histology examination

C57BL/6 mice aged 6-8 weeks were randomly assigned to three groups consisting of three mice each. Two of the groups were designated as experimental and received an intravenous injection of either ^{68}Ga-$NaGdF_4$ or ^{68}Ga-$NaGdF_4$@RGD nanoparticle solution (18.5 MBq, 100 μL) at a dose of 0.1 mmol/kg of Gd^{3+} per mouse. The control group mice were injected with an equivalent volume of PBS solution. On the seventh day after injection, we collected blood samples from all groups through orbital bleeding to perform a complete blood count and biochemical parameter analysis.

Organ histopathology

C57BL/6 mice aged 6-8 weeks were randomly assigned to three groups consisting of three mice each. Two of the groups were designated as experimental and received an intravenous injection of either ^{68}Ga-$NaGdF_4$ or ^{68}Ga-$NaGdF_4$@RGD nanoparticle solution (18.5 MBq, 100 μL) at a dose of 0.1 mmol/kg of Gd^{3+} per mouse. The control group mice were injected with an equivalent volume of PBS solution. Two weeks after injection, the hearts, lungs, livers, kidneys, and spleens of the mice from all three groups were dissected and extracted for Hematoxylin and Eosin (H&E) staining.

Additional Figures

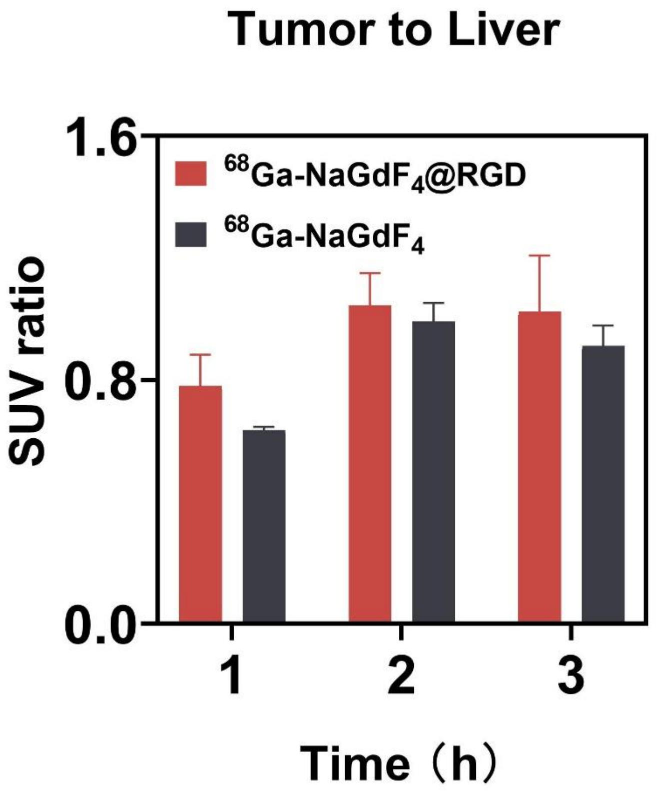

Figure S1. The ratio of SUV_{max} between tumor sites and liver in Hepa 1-6 subcutaneous tumor-bearing mice at various times after injection of ^{68}Ga-$NaGdF_4$@RGD or ^{68}Ga-$NaGdF_4$.

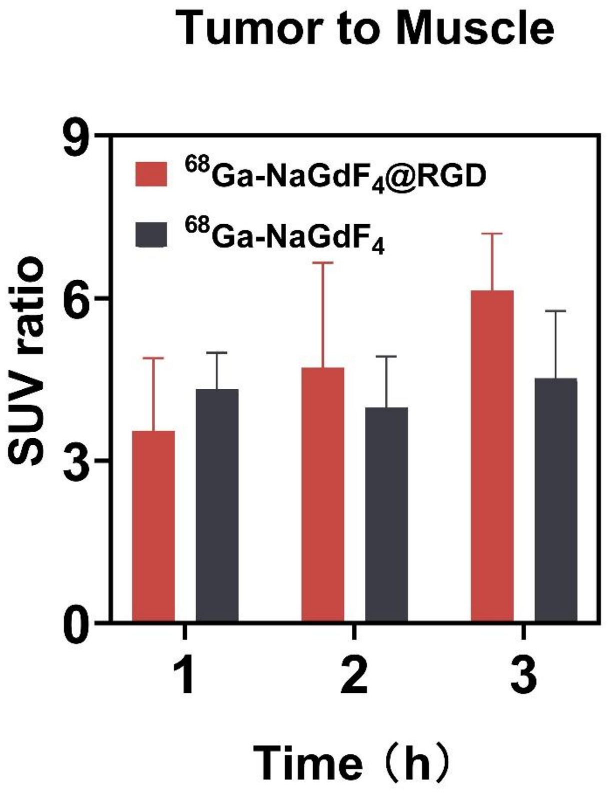

Figure S2. The ratio of SUV_{max} between tumor sites and muscle in Hepa 1-6 subcutaneous tumor-bearing mice at various times after injection of ^{68}Ga-$NaGdF_4$@RGD or ^{68}Ga-$NaGdF_4$.

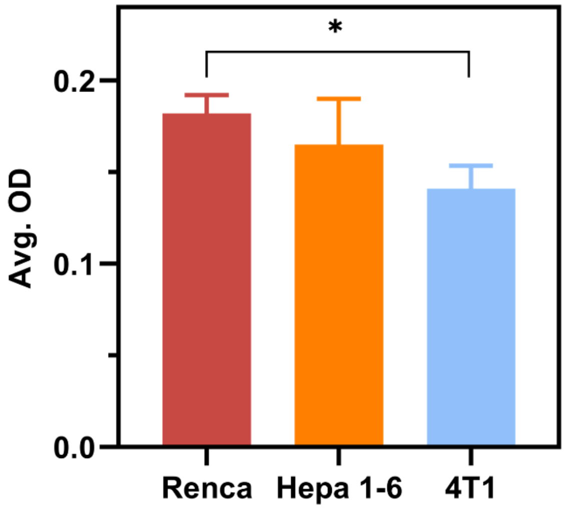

Figure S3. OD of three mouse cell lines co-incubated with $NaGdF_4$@RGD nanoprobes for 12 h, stained with chlorophosphonazo (III). * $P < 0.05$; ** $P < 0.01$; *** $P < 0.001$.

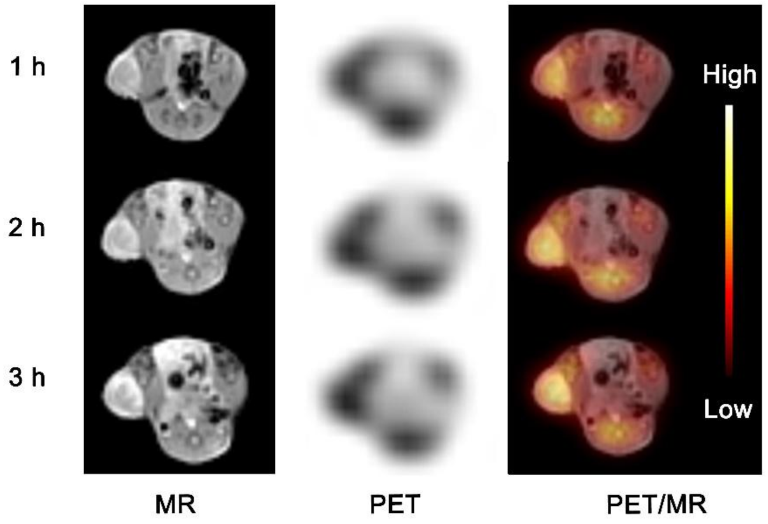

Figure S4. Post-injection MRI, PET, and fused images of the tumor site (right hind limb) in Renca subcutaneous tumor-bearing mice at various times after injection of ^{68}Ga-$NaGdF_4$@RGD.

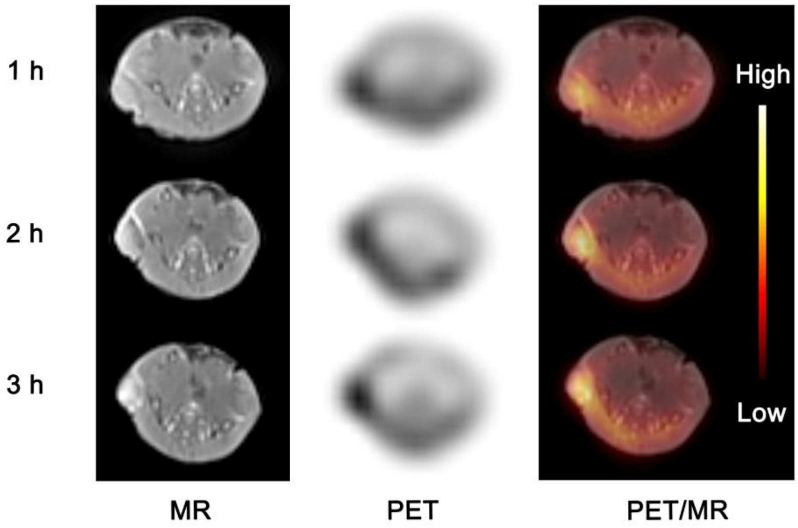

Figure S5. Post-injection MRI, PET, and fused images of the tumor site (right hind limb) in Hepa 1-6 subcutaneous tumor-bearing mice at various times after injection of ^{68}Ga-$NaGdF_4$@RGD.

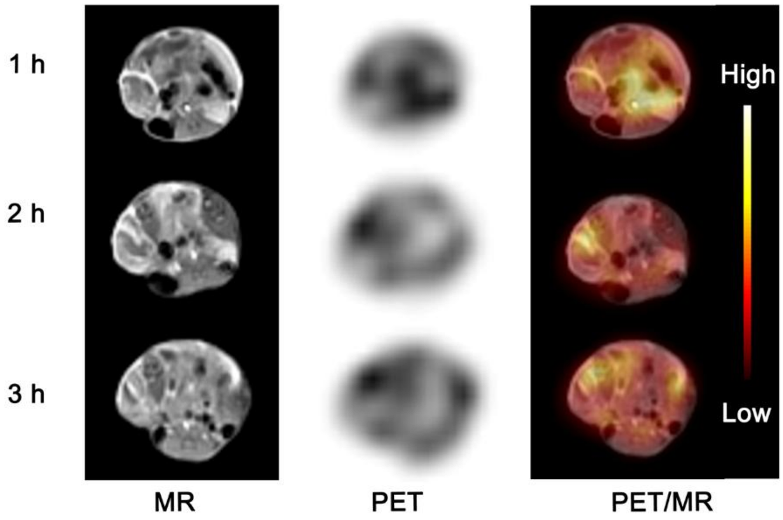

Figure S6. Post-injection MRI, PET, and fused images of the tumor site (right hind limb) in 4T1 subcutaneous tumor-bearing mice at various times after injection of ^{68}Ga-$NaGdF_4$@RGD.

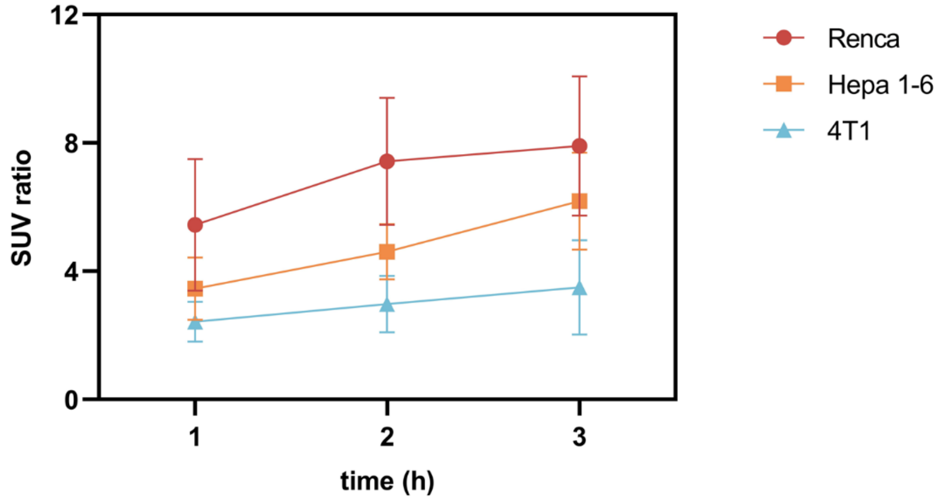

Figure S7. The ratio of SUV_{max} between tumor sites and muscle in Renca, Hepa 1-6, and 4T1 subcutaneous tumor-bearing mice at various times after injection of ^{68}Ga-$NaGdF_4$@RGD.

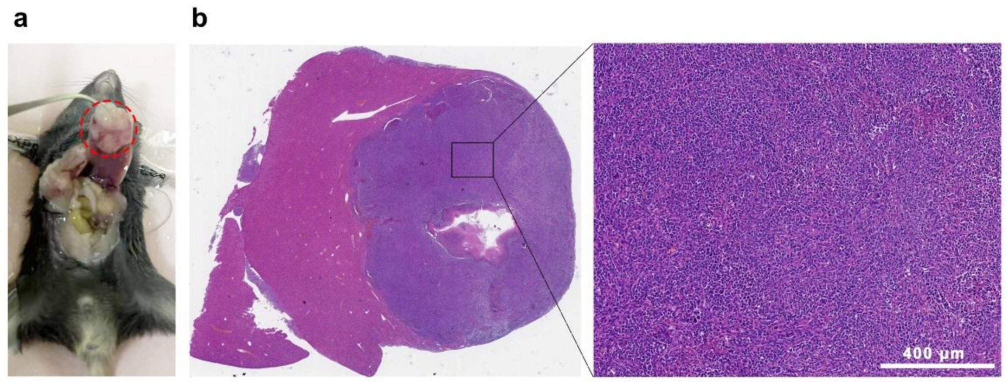

Figure S8. Hepa 1-6 orthotopic liver cancer mouse model. (a) Anatomical photograph of the orthotopic liver cancer mouse model, with the tumor indicated within a red circle; (b) H&E staining of the orthotopic tumor and adjacent cancerous tissue; (c) H&E staining of the orthotopic tumor (100×).

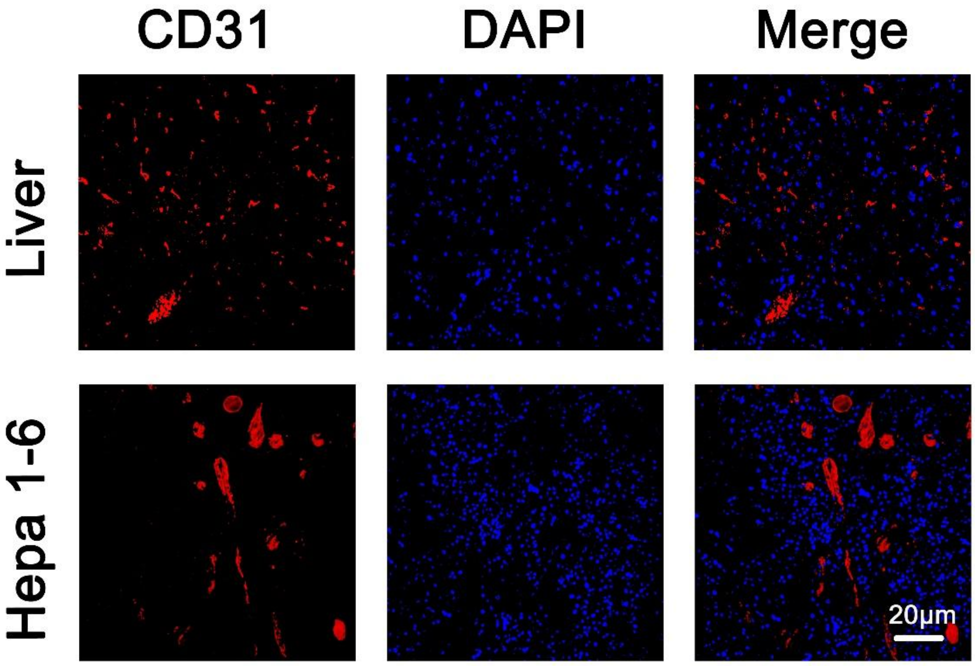

Figure S9. CD31 immunofluorescence staining of orthotopic tumor and normal liver tissue sections in Hepa 1-6 orthotopic liver cancer mice.

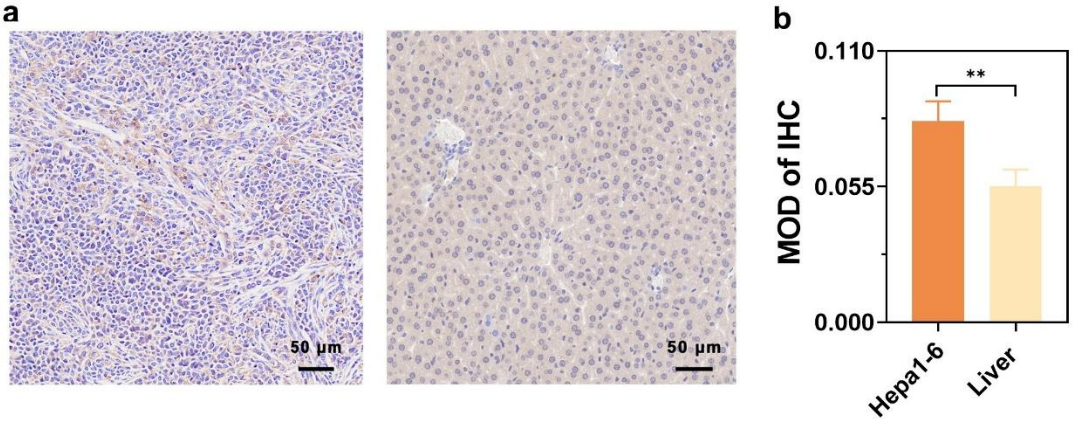

Figure S10. Immunohistochemistry of integrin $\alpha_v\beta_3$ in orthotopic tumor (left) and normal liver tissue (right) sections from Hepa 1-6 orthotopic liver cancer mice. (b) Quantitative analysis of the Immunohistochemistry positive area of integrin $\alpha_v\beta_3$ in orthotopic tumor and normal liver tissue sections of Hepa 1-6 orthotopic liver cancer mice. * $P < 0.05$; ** $P < 0.01$; *** $P < 0.001$.

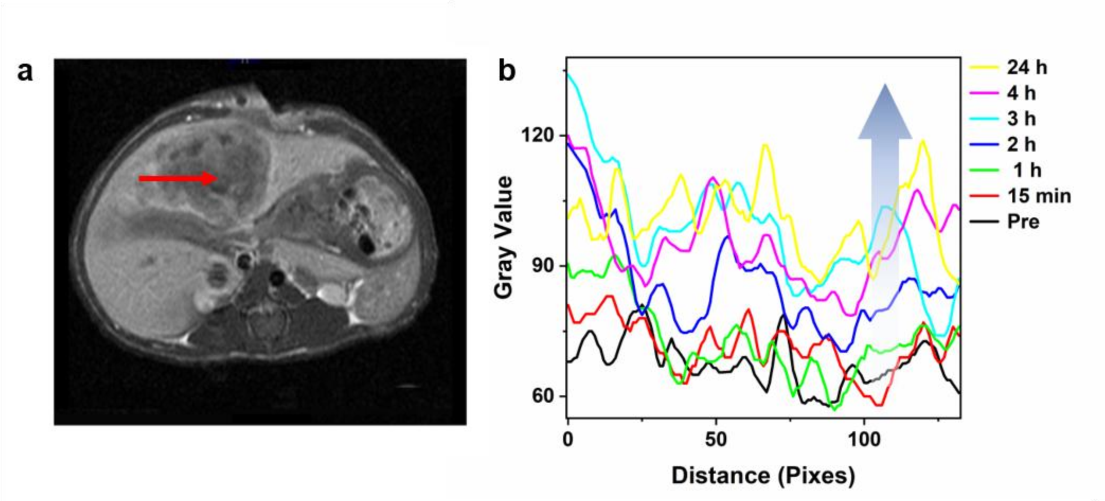

Figure S11. (a) Schematic diagram of tumor progression from periphery to core. (b) Temporal evolution of the MR signal intensity gradient of liver tumor is reflected through the line spectra drawn from the tumor periphery to the core.

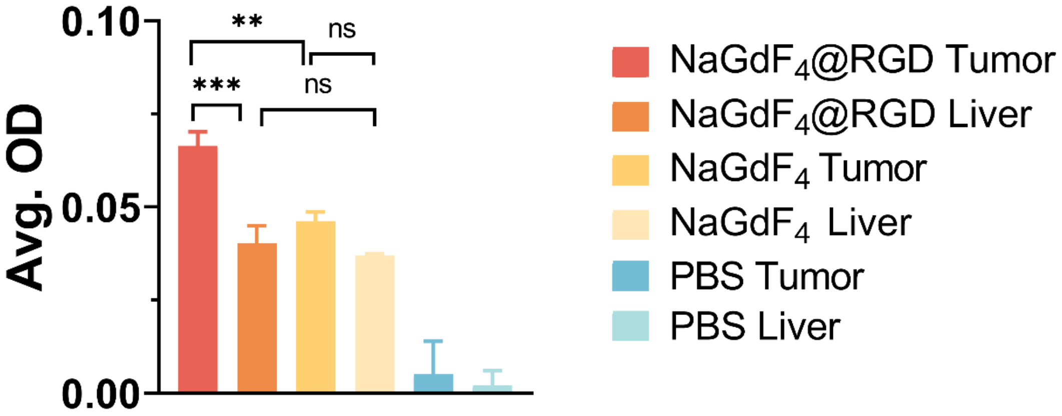

Figure S12. The average OD of chlorophosphonazo (III) staining in orthotopic tumor and normal liver tissue sections 4 h after injection of PBS, $NaGdF_4$, or $NaGdF_4$@RGD nanoprobes in Hepa 1-6 orthotopic liver cancer mice. * $P < 0.05$; ** $P < 0.01$; *** $P < 0.001$.
